# Supplementary material for: Facilitators and barriers impacting in-hospital Trauma Quality Improvement Program (TQIP) implementation across country income levels: a scoping review
Source: BMJ Open. 2023 Feb 17;13(2):e068219. doi: 10.1136/bmjopen-2022-068219 (PMC9944272; doi:10.1136/bmjopen-2022-068219)
Supplement: Supplementary data [file bmjopen-2022-068219supp001.pdf]

Online Supplement:

Online Supplement 1: Database search strategy according to Population, Concept, Context (PCC) framework

**Population:**		
**#1**	**MeSH terms:**	Humans and Trauma Centres
**#2**	**Free Text:**	Population OR populations OR patient OR patients OR adult OR adults OR injured OR victim OR victims OR trauma centre OR trauma centres OR trauma department OR trauma departments OR trauma facility OR trauma facilities OR acute care centre OR acute care centres OR acute care department OR acute care departments OR acute care facility OR acute care facilities OR primary care centre OR primary care centres OR primary care department OR primary care departments OR primary care facility OR primary facilities OR emergency centre OR emergency centres OR emergency department OR emergency departments OR emergency facility OR emergency facilities
**#3**	**#1 OR #2**	
**Concept:**		
**#4**	**MeSH terms:**	Hospital and quality improvement
**#5**	**Free Text:**	Hospital OR hospitals OR in-hospital OR hospital-based OR facility OR facilities AND quality improvement OR quality indicator OR facilitator OR facilitators OR opportunity OR opportunities OR advantage OR advantages OR solution OR solutions OR barrier OR barriers OR challenge OR challenges OR disadvantage OR disadvantages OR inhibitor OR inhibitors OR implementation OR intervention OR innovation
**#6**	**#4 OR #5**	
**Context:**		
**#7**	**MeSH terms:**	Developing countries and developed countries
**#8**	**Free Text:**	Developing country OR developing countries OR developing nation OR developing nations OR developing economy OR developing economies OR low income country OR low income countries OR low income nation OR low income nations OR low income economy OR low income economies OR lower middle income

		country OR lower middle income countries OR lower middle income nation OR lower middle income nations OR lower middle income economy OR lower middle income economies OR upper middle income country OR upper middle income countries OR upper middle income nation OR upper middle income nations OR upper middle income economy OR upper middle income economies OR middle income country OR middle income countries OR middle income nation OR middle income nations OR middle income economy OR middle income economies OR transitional country OR transitional countries OR transitional nation OR transitional nations OR transitional economy OR transitional economies OR developed country OR developed countries OR developed nation OR developed nations OR developed economy OR developed economies OR high income country OR high income countries OR high income nation OR high income nations OR high income economy OR high income economies
**#9**	**#7 OR #8**	
**#10**	**#3 AND #6 OR #9**	3923 Results
**Filter**	English language studies published from June 2009 onwards	

Online Supplement 2: Characteristics of extracted studies

Income Level	Continents/Countries	Source	Study Type	Intervention Type	Intervention Duration (months)
L	Malawi	Chokotho et al. 2019 [16]	Qualitative - Survey	T.R	N/A
L	Malawi	Croke et al. 2020 [17]	Qualitative - Survey	T.R	N/A
L	Mozambique	Hamadani et al. 2019 [18]	Qualitative - Interview	T.R	12
L	Tanzania[a]	Sawe et al. 2020 [19]	Qualitative - Focus Group Discussion	T.R	N/A
L	Tanzania[a]	Sawe et al. 2020 [20]	Qualitative - Interview	T.R	N/A
LM	Pakistan	Hashmi et al. 2013 [21]	Quantitative - logistic regression	T.R, Q.A.P	108
LM	Pakistan	Mehmood et al. 2013 [22]	Qualitative - Survey	T.R	3

LM	Asia: 7	Stelfox et al. 2012 [23]	Mixed Methods	Range - M&M, PDP, AF, QOC Audit, Statistical Analysis	N/A
LM	Kenya	Stevens et al. [24]	Qualitative - Survey	T.R	N/A
UM	Thailand	Fuangworawong et al. 2016 [25]	Qualitative - Survey	Range - M&M, T.R, A.F, PDP	N/A
UM	Peru	LaGrone et al. 2017 [10]	Mixed methods	Range - M&M/T.R	N/A
UM	Argentina	Monteverde et al. 2021 [26]	Qualitative - Univariate/Bivariate descriptive analysis	T.R	114
UM	Brazil	Parreira et al. 2015 [27]	Qualitative - Survey	T.R	12
UM	South Africa	Schuurman et al. 2011 [28]	Qualitative - Survey	T.R	1
UM	Fiji	Wainiqolo et al. 2013 [29]	Qualitative - Survey	T.R	5
UM	Africa: 1, Asia: 1, Europe: 1	Zhou et al. 2021 [30]	Literature Review	T.R	N/A
H	U.K	Hollinshead et al. 2019 [31]	Qualitative - Survey	Educational - B.S.M.P	12
H	Australia	Murphy et al. 2019 [32]	Qualitative - Survey	Multidisciplinary TTT	N/A
H	U.S.A	Newcomb et al. 2020 [33]	Qualitative - Interview	I.P.P	N/A
H	Netherlands	Wiertsema et al. 2021 [34]	Mixed Methods	TTCM	N/A
Range - LMIC	Africa: 6, Asia: 3, South America: 3, North America: 1	Rosenkrantz et al. 2021 [35]	Qualitative - Survey	T.R	Range - 3 = <24 1 = 24-60, 7 = 72-120, 2 = >120
Range - LMIC	Africa: 9, Asia: 4, South	St-Louis et al. 2018 [36]	Literature Review	T.R	N/A

	America: 3, North America: 1, Oceania: 1				
Range - LM/UM	South America: 4	LaGrone et al. 2017 [37]	Qualitative - Survey	Range - M&M, T.R, A.F, Autopsies	N/A
Range - UM/H	South Africa/Canada	Zargaran et al. 2014 [38]	Qualitative - Survey	T.R	1
Range - All	Africa: 6, Asia: 6, North America: 3, South America: 2, Europe: 1, Oceania: 1	Bommakanti et al. 2018 [39]	Literature Review	T.R	N/A
Range - All	Africa: 8, Asia: 6, Europe: 3, North America: 3, Oceania: 2	O'Reilly et al. 2016 [40]	Qualitative - Interview	T.R	N/A
Range - All	Asia: 5, Africa: 3, South America: 1, North America: 1, Oceania: 1	Wild et al. 2020 [41]	Qualitative - Interview	TCC	24
Range - Unspecified	Africa: 7, Asia: 3, North America: 2, Europe: 1, Oceania: 1	Rosenkrantz et al. 2020 [42]	Literature Review	T.R	N/A

Note. L = Low Income, LM = Lower Middle Income, UM = Upper Middle Income, H = High Income, T.R = Trauma Registry, Q.A.P = Quality Assurance Program, M&M = Mortality & Morbidity Conferences, PDP = Preventable Death Panel, A.F = Audit Filter, QOC = Quality of Care, B.S.M.P = Bridges Self-Management Programme, TTT = Trauma Team Training, I.P.P = Injury Prevention Program, TTCM = Transmural Trauma Care Model, TCC = Trauma Care Checklist.

"Qualitative - Survey" design included studies that conducted questionnaires

[a]Tanzania reclassified from low to lower middle income country after data collection of studies
