# Supplementary material for: Facilitators and barriers impacting in-hospital Trauma Quality Improvement Program (TQIP) implementation across country income levels: a scoping review
Source: BMJ Open. 2023 Feb 17;13(2):e068219. doi: 10.1136/bmjopen-2022-068219 (PMC9944272; doi:10.1136/bmjopen-2022-068219)
Supplement: Author's manuscript [file bmjopen-2022-068219.draft_revisions.pdf]

BMJ Open is committed to open peer review. As part of this commitment we make the peer review history of every article we publish publicly available.

When an article is published we post the peer reviewers' comments and the authors' responses online. We also post the versions of the paper that were used during peer review. These are the versions that the peer review comments apply to.

The versions of the paper that follow are the versions that were submitted during the peer review process. They are not the versions of record or the final published versions. They should not be cited or distributed as the published version of this manuscript.

BMJ Open is an open access journal and the full, final, typeset and author-corrected version of record of the manuscript is available on our site with no access controls, subscription charges or pay-per-view fees (http://bmjopen.bmj.com).

If you have any questions on BMJ Open's open peer review process please email info.bmjopen@bmj.com

BMJ Open

Facilitators and barriers impacting in-hospital Trauma Quality Improvement Program (TQIP) implementation A scoping review on the implementation of TQIPs across income levels

Journal:	*BMJ Open*
Manuscript ID	bmjopen-2022-068219
Article Type:	Original research
Date Submitted by the Author:	12-Sep-2022

Complete List of Authors:	Kapanadze, George; Karolinska Institute, Global Public Health<br>Berg, Johanna; Karolinska Institute, Global Public Health; Skånes universitetssjukhus Malmö, Emergency and Internal medicine<br>Sun, Yue; Karolinska Institute, Global Public Health<br>Gerdin Wärnberg, Martin ; Karolinska Institutet, Department of Global Public Health; Karolinska University Hospital, Function Perioperative Medicine and Intensive Care

Keywords:	Quality in health care < HEALTH SERVICES ADMINISTRATION & MANAGEMENT, TRAUMA MANAGEMENT, Change management < HEALTH SERVICES ADMINISTRATION & MANAGEMENT

SCHOLARONE™ Manuscripts

I, the Submitting Author has the right to grant and does grant on behalf of all authors of the Work (as defined in the below author licence), an exclusive licence and/or a non-exclusive licence for contributions from authors who are: i) UK Crown employees; ii) where BMJ has agreed a CC-BY licence shall apply, and/or iii) in accordance with the terms applicable for US Federal Government officers or employees acting as part of their official duties; on a worldwide, perpetual, irrevocable, royalty-free basis to BMJ Publishing Group Ltd ("BMJ") its licensees and where the relevant Journal is co-owned by BMJ to the co-owners of the Journal, to publish the Work in this journal and any other BMJ products and to exploit all rights, as set out in our licence.

The Submitting Author accepts and understands that any supply made under these terms is made by BMJ to the Submitting Author unless you are acting as an employee on behalf of your employer or a postgraduate student of an affiliated institution which is paying any applicable article publishing charge ("APC") for Open Access articles. Where the Submitting Author wishes to make the Work available on an Open Access basis (and intends to pay the relevant APC), the terms of reuse of such Open Access shall be governed by a Creative Commons licence – details of these licences and which Creative Commons licence will apply to this Work are set out in our licence referred to above.

Other than as permitted in any relevant BMJ Author's Self Archiving Policies, I confirm this Work has not been accepted for publication elsewhere, is not being considered for publication elsewhere and does not duplicate material already published. I confirm all authors consent to publication of this Work and authorise the granting of this licence.

For peer review only

Facilitators and barriers impacting in-hospital Trauma Quality Improvement Program (TQIP) implementation

A scoping review on the implementation of TQIPs across income levels

George Kapanadze, MMSc; Johanna Berg, MD; Yue Sun, MPH; Martin Gerdin Wärnberg MD, PhD

Author Affiliations: Department of Global Public Health, Karolinska Institutet, Solna, Sweden (Kapanadze); Department of Global Public Health, Karolinska Institutet, Solna, Sweden, Department of Internal and Emergency Medicine, Skåne University Hosiptal, Malmö (Berg); Department of Global Public Health, Karolinska Institutet, Solna, Sweden (Sun); Department of Global Public Health, Karolinska University Hospital, Karolinska Institutet, Solna, Sweden (Wärnberg).

Corresponding Author: George Kapanadze, MMSc, Department of Global Public Health, Karolinska Institutet,Widerströmska huset Tomtebodavägen 18 A, plan 3, 171 65 Solna, Sweden (george.kapanadze@stud.ki.se)

For peer review only

Abstract:

Introduction: Trauma describes physical injury along with the body's associate response and is a leading cause of mortality and morbidity globally, with low and middle income countries (LMICs) disproportionately affected. Understanding the implementation of in-hospital Trauma Quality Improvement Programs (TQIPs) and the factors' determining success is critical to reduce the global trauma burden. The purpose of the review was to identify key facilitators and barriers to TQIP implementation across income levels by evaluating the range of literature on the topic.

Methods: We used the information sources PubMed, Web of Science, and Global Index Medicus. The eligibility criteria were English language studies, of any design, published from June 2009 - January 2022. The Preferred Reporting Items of Systematic Reviews and Meta-Analyses checklist extension for scoping reviews were used to carry out a three-stage screening process. Content analysis using the Consolidated Framework for Implementation Research (CFIR) identified facilitator and barrier themes for in-hospital TQIP implementation.

Results: Twenty-eight studies met the eligibility criteria from 3923 studies. The main facilitators and barriers identified were the need to prioritise staff education and training, strengthen dialogue with stakeholders, and provide standardised best-practice guidelines. Data quality improvements were more apparent in LMICs while high-income countries (HICs) emphasised increased communication training.

Conclusions: Stakeholder prioritisation of in-hospital TQIPs, along with increased knowledge and consensus on trauma care best practice will further advance efforts to lower the global trauma burden. The focus of future in-hospital TQIPs in LMICs should primarily be concerned with improving data quality of registries, while interventions in HICs should focus on communication skills of healthcare professionals.

For peer review only - http://bmjopen.bmj.com/site/about/guidelines.xhtml

Key Questions:

What is already known?

- The development of in-hospital Trauma Quality Improvement Programs (TQIPs) has steadily risen, particularly in high income countries, but limited attention has been placed on identifying factors that impact implementation success across multiple contexts.

What does this study add?

- The study, to the best of our knowledge, is the first evidence synthesis identifying facilitators and barriers for in-hospital TQIP implementation across income levels.
- Facilitators and barriers to in-hospital TQIPs were similar across all income levels, particularly regarding staff education, stakeholder engagement, and standardisation of guidelines.
- A need for data quality improvements was prevalent in LMICs while high-income studies specified communication training as an area of improvement.

What do the new findings imply?

- More studies should focus on cross-collaboration of in-hospital TQIPs as the factors determining implementation success are similar regardless of income level.
- There is a need for more research to prioritise data quality improvements in trauma registry development in LMICs as a first step in the in-hospital TQIP cycle.

For peer review only

Introduction:

Trauma is the clinical entity composed of physical injury and the body's associated response [1]. Trauma is a leading cause of death and disability worldwide **[2]**. To improve in-hospital care, multiple Trauma Quality Improvement Programs (TQIPs) have been developed, typically originating in high-income countries (HICs). Health system changes in care for trauma patients have been attributed to significant improvements in care quality and outcomes in HICs [3].

A recent systematic review in HICs showed that the management of patients in mature trauma systems was associated with decreased mortality outcomes compared to patients in non-trauma systems [4]. Thus, further research is needed to assess trauma systems in low and middle income countries (LMICs) to inform health system strengthening for trauma care [3].

Quality Improvement (QI) is designed to achieve positive change in a specific process [5], which can be highly dependent on the perspectives, attitudes, and behaviours of patients and healthcare professionals in their local context [6]. Although QI aims to improve the quality of care, organisations often struggle with its implementation [7]. While implementation research has improved significantly, there remain substantial gaps in knowledge and understanding of factors that facilitate or impede efforts to implement QI in health care organisations [8].

Despite research on developing in-hospital TQIPs steadily rising, little context-relevant guidance exists to help policymakers set priorities for implementation [9]. A recent study found a lack of in-depth examination on barriers and facilitators to TQIP implementation in LMICs [10]. Thus, the scoping review aimed to identify key facilitators and barriers to in-hospital TQIP implementation across income levels.

For peer review only - http://bmjopen.bmj.com/site/about/guidelines.xhtml

Methods:

We conducted a scoping review to understand available evidence on factors influencing in-hospital TQIP implementation in accordance with the Preferred Reporting Items for Systematic Reviews and Meta-Analyses extension for Scoping Reviews (PRISMA-ScR) [11]. A scoping review was most appropriate due to the broad nature of the topic along with the wide variety of potential factors impacting quality improvement implementation in differing income settings. The income level of each included country was derived based on World Bank classification at the time of publication [12].

Search Strategy

The search strategy was developed with a librarian using keywords, synonyms, and medical subject headings (MeSH) as shown in online supplement 1**.** The review searched three databases, *PubMed*, *Web of Science* and *Global Index Medicus*, for studies from June 2009 to January 2022.

Study Selection

Given the broad scope of the review all in-hospital TQIPs were considered, along with studies of any design. The screening process was completed by two authors, assisted by a third reviewer. The following inclusion criteria were used:

Any English language studies evaluating facilitators and barriers to implementation of system-wide in-hospital QI programs in trauma populations worldwide.

Studies were excluded if they were:

Non-English, unpublished, if they described projects where implementation was not a focus, or if they discussed non-hospital TQIPs.

Data Charting

Data extraction was performed using a predetermined form addressing study identifying information; geographic location; population and study setting; income level at the time of publication; data collection methods; QI intervention type and duration; implementation facilitators and barriers; implementation outcome and study funding sources. Appropriate quotations were identified and extracted through deductive content analysis.

For peer review only - http://bmjopen.bmj.com/site/about/guidelines.xhtml

Summarising and Reporting Results

Facilitator and barrier selection was guided by the Consolidated Framework for Implementation Research (CFIR) - illustrated in figure 1. The framework is composed of five domains: intervention characteristics, outer setting, inner setting, individual characteristics, and implementation process [13]. These domains provide a practical guide for systematically assessing potential facilitators and barriers to TQIP implementation [14], while offering insights to explain differences across income levels [15].

Patient and Public Involvement:

Patients and the public were not involved in the design and conduct of this research.

For peer review only

Results:

The literature search yielded 3923 studies, 3291 studies passed title and abstract screening while 170 reports were identified for full-text screening. The number of extracted studies was 28, with details on study selection found in figure 2. Most of the studies originated in upper middle income countries (7 [25%]), followed by low income (5 [18%]), high income (4 [14%]), and lower middle income (4 [14%]). The most represented continents in the review were Africa and Asia, with South Africa (11 [39%]) and Pakistan (9 [32%]) appearing most frequently. Most studies solely discussed trauma registries as the prominent in-hospital TQIP (18 [64%]) while a small number evaluated two or more TQIPs in their reports (5 [18%]). Full study characteristic details are found in online supplement 2.

The CFIR domains identified most in the literature were intervention characteristics and inner setting. A summary of facilitators and barriers according to the CFIR are in Tables 1 and 2.

Table 1: Summary of in-hospital TQIP implementation facilitators using the CFIR

CFIR Domain	CFIR Construct	Facilitators	Quotes
Intervention Characteristics	Relative Advantage	Open dialogue with key stakeholders	1. *Active cooperation and extensive input from MoH stakeholders* [17]<br>2. *Appealing to stakeholders higher up in DoH* [35]
	Adaptability	Tailoring intervention to local context	1. *All data collection was carried out by local Mozambican health workers* [18]<br>2. *Participants highlighted that patient care should be adapted to local circumstances* [23]
	Complexity	Focus on simplicity	1. *Balance between sufficient detail and simplicity of the data collection process* [29]<br>2. *Less is more when it comes to trauma registry* [40]

For peer review only - http://bmjopen.bmj.com/site/about/guidelines.xhtml

Outer Setting	Patient needs & Resources	Prioritising patient care	1. *More focus on the everyday things that patients have to deal with* [34]<br>2. *Harnessing…clinical enthusiasm to improve trauma care* [40]
	External policies & incentives	Collaboration with national/internation al bodies	1. *Increased focus on trauma academics through regional and international collaborations* [21]<br>2. *Seek potential collaborators with interest* [35]
Inner Setting	Implementation Climate		
	Learning Climate	Staff education & training	1. *Engage nursing and medical staff in multidisciplinary training* [31]<br>2. *Training needs to include strategies that address fundamental interpersonal communication* [32]
	Readiness for implementation		
	Leadership engagement	Prioritise buy-in	1. *Leadership was identified as having a clear impact on team factors* [32]<br>2. *The IP coordinators' work was influenced by the support from direct managers and senior leadership* [33]
	Available Resources	Diversify resource sources	1. *External training resources, such as national or state conferences, were important to allow IPCs to connect* [27]<br>2. *Broadening the scope of the registry to apply for additional grants* [35]
Characteristics of Individuals	Knowledge & Beliefs about the intervention	Generate understanding of QI	1. *There is a need for increased dissemination of QI programs as a means of empowering local providers to participate in their health systems* [37]

For peer review only

			2. *Formulate very clearly a vision, mission and strategy that everyone understands* [40]
	Other Personal Attributes	Communication training	1. *Important to find ways to sustain changes in clinician communication style* [22]<br>2. *Training needs to include strategies that address fundamental interpersonal communication alongside other solutions* [32]
Process of Implementation	Reflecting & Evaluating	Strengthening data quality	1. *Increasing real and perceived efficacy of M&M conferences…through establishment of standardised case selection criteria* [37]<br>2. *Regular audits and staged validation of data* [40]
	Engaging		
	Champions	Identifying dedicated staff	1. *Sometimes I just get inspired by something that occurs to me as nothing is being done about it currently* [33]<br>2. *Identify a person with passion and zeal to drive the effort* [35]

Table 2: Summary of in-hospital TQIP implementation barriers using the CFIR

CFIR Domain	CFIR Construct	Barriers	Quotes
Intervention Characteristics	Relative Advantage	Limited desire for QI initiatives	1. *A "so what" attitude amongst stakeholders from other nearby facilities* [35]<br>2. *Current lack of motivation for QI programs in Latin America* [37]

For peer review only - http://bmjopen.bmj.com/site/about/guidelines.xhtml

	Adaptability	Resistance to change	1. *Surgeon's preference for autonomy and self-reliance over standardisation: "I do it my way, you do it yours"* [10]<br>2. *AHPs identified the barriers of…'the way things are always done"* [31]
	Complexity	Competing interests between groups	1. *Institutional and national prioritisation of other patient care objectives* [10]<br>2. *Trauma is a very low priority for most clinicians* [40]
Outer Setting	Patient needs & Resources	Staff time constraints	1. *Staff have multiple demands on their time* [17]<br>2. *Surgeon spends less time with a patient, he can proceed with the next patient* [34]
	External policies & incentives	Lack of common guidelines	1. *Absence of uniform policies, guidelines, and protocols* [23]<br>2. *No standards or best practices in place for the collection of data* [42]
Inner Setting	Implementation Climate		
	Learning Climate	Professional hierarchies	1. *Senior staff attempting to maintain the status quo in order to fly under the radar* [10]<br>2. *Limited ability to engage leaders* [23]
	Readiness for implementation		
	Leadership engagement	Lack of buy-in	1. *Some stakeholders…created barriers by prioritising other clinical issues* [35]<br>2. *Rest of the stakeholders and the doctors did not buy-in to this concept of using a registry* [40]

For peer review only - http://bmjopen.bmj.com/site/about/guidelines.xhtml

	Available Resources	Limited Resources	1. *Hospitals cannot afford to hire specialised personnel solely to collect data* [27]<br>2. *Make do with the resources that you have, because the funding will not be there* [40]
Characteristics of Individuals	Knowledge & Beliefs about the intervention	Lack of staff belief in QI interventions	1. *Some initial skepticism from counterparts* [17]<br>2. *It's not fitting into anybody's career plans at this point in time…trauma happens to poor people* [41]
	Other Personal Attributes	Lack of staff knowledge	1. *Almost half of newly-graduated physicians do not have experience working with this QI tool during their training* [25]<br>2. *Not a healthy understanding of how we do our work or what's needed to do our work* [33]
Process of Implementation	Reflecting & Evaluating	Poor Data Quality	1. *Challenges reported included the high rates of missing data* [18]<br>2. *A major hurdle is data quality of clinical records* [26]
	Engaging		
	Champions	Overworked Staff	1. *Human resources to meet volume of data collection were underestimated* [24]<br>2. *Constant suggesting by the faculty to use overworked residents to collect trauma data* [35]

Domain 1: Characteristics of the Intervention

Open dialogue with key stakeholders facilitates the implementation process [10,17,35,36]. A multi-centre digital trauma registry implementation in Malawi revealed that consistent communication between Ministry of Health stakeholders and the research team contributed to the

For peer review only

success of the registry [17]. Competing interests between groups acted as a barrier to establishing communication channels between stakeholders [10,35,40]. The absence of vision for trauma care was highlighted by a custodian from a multi-hospital in a developing country as there was a need to *"show the vision of better trauma care to lots of people, because trauma is a very low priority for most clinicians, and it happens to poor people"* [40 p.119].

Another key theme was the importance of adaptability. A focus on tailoring the intervention to the local context to ensure success and sustainability was apparent [10,18,23,24,32,35,37,38]. The implementation of a multi-hospital injury surveillance program in Mozambique was driven by local data collectors that enhanced the sustainability of the project [18]. Tailoring programs to local conditions is often made difficult by individuals' resistance to change [10,31]. An educational self-management intervention in a trauma centre in the UK revealed that the roll-out of support was hindered by team members preferring to continue with established practices, which limited widespread adoption [31].

Attention to simplicity is an implementation facilitator [19,29,35,40]. A trauma registry development study in LMICs emphasised the significance of keeping design simple through standardisation of variables to enhance accuracy and understanding [35]. Standardisation requires widespread staff and stakeholder support, which is often lacking due to limited desire for QI. A survey of trauma care providers in the Andean region revealed that less than half of respondents *"ever witnessed a change occur in their institution",* which influenced the *"current lack of motivation for QI programs"* [37 p.1992].

Domain 2: Outer Setting

Prioritising patient care during implementation was widely addressed [21,31,34,38]. A Dutch evaluation of the implementation of the Transmural Trauma Care Model found that patients' experience improved as more focus was placed on day-to-day issues patients encountered [34]. However, time constraints were a key barrier to providing improved patient care [10,17,19,24,25,26,31,34]. A Peruvian study on the status of in-hospital TQIPs revealed the

For peer review only - http://bmjopen.bmj.com/site/about/guidelines.xhtml

"prevalence of dual practice" [10 p.966], surgeons working at both public and private institutions, meant that clinicians did not have time to effectively participate in emerging QI initiatives.

Collaboration with national and international bodies was a facilitator to successful implementation [21,35]. A questionnaire with trauma registry stewards in LMICs showed a need to *"develop skills at political lobbying to induce support from the administration*" to provide a platform for long-term aid [35 p.2221]. The lack of common guidelines was highlighted as a barrier [10,26,27,35,40,42]. A study exploring staff trauma experiences in LMICs underlined that organisational diversity across the region led to limited uniform policies which reduced communication among trauma facilities [10].

Domain 3: Inner Setting

Increased staff education and training strengthened the implementation climate of QI interventions [16,18,19,22,23,27,29,31,32,38,40]. An integrated self-management support paper emphasised successful training as accessible to staff at all levels, with a focus on flexibility [31]. Professional hierarchies acted as a barrier to the continued education of all staff [10,23,31,32]. A training program clinician survey found that traditional decision-making hierarchies acted as a major barrier to effective education as team members were *"reluctant to speak up to highlight a problem, clarify information, or question a senior's decision when they were concerned"* [32 p.1151].

Implementation readiness was positively impacted by prioritising leadership buy-in [10,22,29,35,38]. An electronic registry in Pakistan highlighted obtaining senior management buy-in as a catalyst to improving trauma care quality [22]. Lack of stakeholder buy-in acted as a barrier to achieving progress in trauma care [35,40]. A trauma registry custodian from an LMIC hospital stated that a common opinion was that *"even basic care is not being given to the patients...how are we going to sort of spend for a registry?"* [40 p.122].

Diversifying resource sources is fundamental for successful implementation [27,35]. Respondents in a resource-constrained setting highlighted demand should be met by broadening the registry scope to be eligible for more grants [35]. This was in response to a common barrier around limited

For peer review only - http://bmjopen.bmj.com/site/about/guidelines.xhtml

resources [18,19,21,22,27,28,35,40]. A trauma registry custodian explained that staff must *"make do with the resources that you have, because the funding will not be there"* [40 p.120].

Domain 4: Characteristics of Individuals

Generating staff understanding of QI is needed to encourage change [10,29,40]. Increased QI dissemination can help develop greater public awareness [10,29]. A barrier to this effort is the lack of staff belief in QI interventions [10,17,35,41]. A participant from a healthcare provider interview on implementing the World Health Organisation (WHO) TCC highlighted that *"nobody is really trying to do it as a career"* as the majority *"would rather be doing joint replacements, rather than worrying about the poor people falling off trains and motorcycles"* [41, p.19].

Communication training was a facilitator for TQIP implementation [17,24,31,32]. A trauma training clinician survey emphasised developing interpersonal staff communication alongside strengthening emergency communication methods [32]. Limited staff knowledge acts as a hurdle to improved communication [10,24,25,33]. A subject from a US injury prevention program highlighted the lack of healthy understanding of QI requirements as they are viewed as *"the black sheep"* of the department, making it a constant struggle to *"seek out other mentors of people that do similar work"* [33 p.339].

Domain 5: Process of Implementation

Identifying staff to act as a champion for the QI intervention is significant [19,33,35,41]. Trauma registry questionnaire respondents felt that limited stakeholder engagement could be overcome by having a champion to drive and mobilise buy-in [35]. Identifying a champion is made difficult by the barrier of overworked staff [10,17,18,20,24,25,35]. A Tanzanian hospital was so understaffed that *"you will find you have one doctor at night and ten patients show up at once"*, making it difficult to document records and motivate staff [20 p.26].

Strengthening data quality was highlighted as a facilitator [16,17,22,23,28,29,40]. Asia-Pacific trauma leaders proposed establishing standardised minimum data requirements to collect more

For peer review only - http://bmjopen.bmj.com/site/about/guidelines.xhtml

complete patient information and integrate injury surveillance with data collection [23]. These efforts were hindered by poor data quality across income levels [16,18,21,23,26,35,40]. An Argentinian registry implementation project found a large variation in trauma admission protocols and widespread under-reporting, impacting the quality of clinician records [26].

Common facilitators and barriers were arranged by income class and are summarised in table 3.

Table 3: Main facilitators and barriers to TQIP implementation by income level

Income Class	Facilitators	Barriers
L	• Staff education & training<br>• Open dialogue with key stakeholders<br>• Strengthening data quality	• Poor Data Quality<br>• Limited Resources<br>• Overworked Staff
LM	• Staff education & training<br>• Tailoring to local context<br>• Strengthening data quality	• Poor data quality<br>• Lack of buy-in<br>• Lack of common guidelines
UM	• Staff education & training<br>• Tailoring to local context<br>• Prioritising buy-in	• Poor data quality<br>• Lack of common guidelines<br>• Lack of staff knowledge
H	• Staff education & training<br>• Communication training<br>• Prioritising patient care	• Professional hierarchies<br>• Lack of common guidelines<br>• Overworked staff

For peer review only - http://bmjopen.bmj.com/site/about/guidelines.xhtml

Discussion:

In-hospital TQIP facilitators and barriers were similar across all income levels. Facilitators and barriers, grouped under the five CFIR domains, identified prioritising staff education and training, strengthening dialogue with stakeholders, and increasing standardised guidelines for best practice as key facilitators going forward. Major barriers were the need to prioritise data quality improvement in LMICs and improved communication training in HICs. Studies focusing on in-hospital TQIPs in LMICs were limited to a few countries, indicating that the geographic scope of quality improvement efforts must be widened in these regions.

A facilitator was strengthening stakeholder dialogue to increase buy-in for in-hospital TQIPs. This supports a previous qualitative interview study that proposed adopting a hybrid QI model where experts would set basic guidelines for programs, incorporating feedback from frontline staff members according to local conditions [43]. Stakeholder dialogue barriers were a limited desire for TQIPs, resistance to change, and competing group interests. Studies reported the need to tailor interventions to the local context, along with focusing on simplicity to improve communication. An earlier grounded theory analysis of HIC interviews identified important strategies to improve stakeholder engagement as clarity of purpose, inviting participation, and engaging clinicians with feedback [44].

A reported barrier across all income levels was the lack of common guidelines throughout the trauma care timeline. A review on LMIC trauma registry implementation highlighted that the WHO's *Injury Surveillance Guidelines* is only focused on collecting data on injury events rather than also addressing hospital care or trauma outcomes [45]. The uncoordinated and resource-intensive nature of data collection acted as a barrier that contributed to staff time constraints. A survey revealed that there is no single resource available that provides comprehensive insight into experiences across trauma registries [46]. To overcome this, QI guideline standardisation was emphasised as a facilitator to improve the success of in-hospital implementation.

Limited in-hospital TQIP durability data underlines the need to prioritise research on sustainable interventions. An LMIC scoping review on trauma registry implementation highlighted the need to prioritise sustainable registry development as a significant step forward [45]. Key barriers

For peer review only - http://bmjopen.bmj.com/site/about/guidelines.xhtml

reported were the lack of long-term funding and limited buy-in for QI initiatives. Increased dissemination of results is a critical facilitator to establish prolonged TQIP support, through enhancing engagement and QI understanding. A trauma care provider survey in the Andean region concluded that increased QI dissemination can empower local providers to participate in health systems and drive change [37]. The increase in peer-reviewed QI reports from LMICs emphasises dissemination through publications as a viable means of providing guidance for future programs and expanding QI knowledge [47].

Education and training is an essential facilitator to improve the chances of successful implementation. This was mentioned at all income levels, specifically in terms of interpersonal communication in HICs. Enhancing communication channels between trauma teams to improve health outcomes is increasingly common in HICs. A quantitative study found that well-defined interdisciplinary communication between the trauma service and surgical specialists reduced the time-to-operation for facial trauma patients [48]. Common barriers were limited staff belief and knowledge in QI interventions. While elements of the WHO's guidelines for TQIPs have been mandated by governments across income levels [49], there have not been clear protocols put into place for implementing, evaluating, and sustaining interventions. This makes it difficult to provide standardised training for staff, which can impact the quality of patient care.

Enhancing data quality is a crucial facilitator to strengthening in-hospital implementation. Trauma registries were the most common in-hospital TQIP intervention, particularly in LMICs, with studies citing poor data quality as the main barrier. Reasons for the lack of data quality centred on high rates of missing data and limited standardised variables for comparison. A scoping review assessing trauma registry data quality found that only four out of sixty-nine publications provided a general classification of data quality, in which the taxonomy was inconsistent [50]. A similar systematic review identified only ten studies that evaluated data quality in trauma registries [51]. The majority assessed data quality based on completeness, with large differences reported between papers, and no evidence found on data precision or timeliness [51]. This lack of uniformity makes it difficult to draw insights on the quality of trauma care delivered and compare across income levels. The Lancet Global Health Commission on High Quality Health Systems underlined the

For peer review only - http://bmjopen.bmj.com/site/about/guidelines.xhtml

disparity between the growing global injury burden and the limited data availability on care quality [52].

Strengths and Limitations:

The review covered a broad range of TQIP interventions and included studies with any design. The screening process was conducted along with a peer-reviewer, which allowed for comparison and greater input in decision-making. This review provides a snapshot of the research field through a methodologically robust process which includes a transparent method of reviewing in a limited timeframe [53]. The review captured a large number of studies for analysis from various databases.

The use of three databases and the restriction of literature to English-language studies could have biased the findings if excluded studies were significantly different to those included in the scoping review. The use of more databases, a wider timeframe, and the inclusion of a greater number of studies could have provided data that may have been neglected. We attempted to limit the selection bias by consulting a librarian with extensive experience conducting scoping reviews when selecting databases.

Grouping literature into income classes despite differing country contexts may provide another limitation. The scoping review process does not place as much emphasis on assessing the quality of included studies when compared with systematic reviews [54]. The lack of quality assessment provides a risk of including studies with lesser quality. There is a risk of researcher bias as the process of choosing and analysing literature can be subjective. Important steps to avoid researcher bias were focusing on the research question, following the eligibility criteria, and cross-referencing screening selection with a peer–reviewer.

Conclusion:

Staff education and training, strengthening stakeholder dialogue, and increasing standardised trauma care guidelines were reported as the main facilitators, while major barriers identified were poor data quality in LMICs and the lack of effective communication training in HICs. Stakeholder

For peer review only - http://bmjopen.bmj.com/site/about/guidelines.xhtml

prioritisation of TQIPs, along with increased knowledge and consensus on trauma care best practice will further advance efforts to lower the global trauma burden. The focus of future in-hospital TQIPs in LMICs should primarily be concerned with improving the data quality of registries, while interventions in HICs should focus on the communication skills of healthcare professionals.

List of abbreviations:

Abbreviation	Meaning
LMIC	Low and middle income country
TQIP	Trauma Quality Improvement Program
CFIR	Consolidated Framework for Implementation Research
HIC	High income country
QI	Quality improvement
WHO	World Health Organisation

Figure Legends:

Figure 1: CFIR Conceptual Framework Outline adapted from Damschroder et al. 2009 [13]

Figure 2: Study selection according to PRISMA ScR statement

For peer review only

For peer review only - http://bmjopen.bmj.com/site/about/guidelines.xhtml

References:

1. Gerdin M. The risk of dying: predicting trauma mortality in urban Indian hospitals [dissertation on the internet]. Stockholm: Karolinska Institutet; 2015 [cited 2022 Mar 23]. Available from: https://openarchive.ki.se/xmlui/bitstream/handle/10616/44832/Thesis_Martin_Gerdin.pdf
2. Vos T, Lim SS, Abbafati C, Abbas KM, Abbasi M, Abbasifard M, et al. Global burden of 369 diseases and injuries in 204 countries and territories, 1990–2019: a systematic analysis for the Global Burden of Disease Study 2019. Lancet. 2020 Oct 17;396(10258):1204-22.
3. Whitaker J, Denning M, O'Donohoe N, Poenaru D, Guadagno E, Leather A, et al. Assessing trauma care health systems in low-and middle-income countries, a protocol for a systematic literature review and narrative synthesis. Syst. Rev. 2019 Dec;8(1):1-6.
4. Alharbi RJ, Shrestha S, Lewis V, Miller C. The effectiveness of trauma care systems at different stages of development in reducing mortality: a systematic review and meta-analysis. World J of Emerg Surg. 2021 Dec;16(1):1-2.
5. Itri JN, Bakow E, Probyn L, Kadom N, Duong PA, Gettle LM, et al. The science of quality improvement. Acad Radiol. 2017 Mar;24(3):253-62.
6. Pope C, Van Royen P, Baker R. Qualitative methods in research on healthcare quality. Qual Saf Health Care. 2002 Jun;11(2):148-52.
7. Alexander JA, Weiner BJ, Shortell SM, Baker LC, Becker MP. The role of organizational infrastructure in implementation of hospitals' quality improvement. Hosp Top. Winter 2006;84(1):11-21.
8. Alexander JA, Hearld LR. The science of quality improvement implementation: developing capacity to make a difference. Med care. 2011 Dec; 49 Suppl:S6-20.
9. Reynolds TA, Stewart B, Drewett I, Salerno S, Sawe HR, Toroyan T, et al. The impact of trauma care systems in low-and middle-income countries. Annu Rev Public Health. 2017 Mar 20;38:507-32.

For peer review only - http://bmjopen.bmj.com/site/about/guidelines.xhtml

10. *LaGrone LN, Fuhs AK, Egoavil EH, Rodriguez Castro MJ, Valderrama R, IsquithDicker LN, et al. Mixed-methods assessment of trauma and acute care surgical quality improvement programs in Peru. World J Surg. 2017 Apr;41(4):963-969.*
11. Tricco AC, Lillie E, Zarin W, O'Brien KK, Colquhoun H, Levac D,et alS. PRISMA extension for scoping reviews (PRISMA-ScR): checklist and explanation. Ann Intern Med. 2018 Oct 2;169(7):467-473.
12. WDI - The World by Income and Region [Internet]. Datatopics.worldbank.org. 2022 [cited 2022 April 25]. Available from: https://datatopics.worldbank.org/world-development-indicators/the-world-by-income-and-region.
13. Damschroder LJ, Aron DC, Keith RE, Kirsh SR, Alexander JA, Lowery JC. Fostering implementation of health services research findings into practice: a consolidated framework for advancing implementation science. Implement Sci. 2009 Aug 7;4:50
14. CFIR Research Team-Center for Clinical Management Research. Consolidated Framework for Implementation Research [Internet]. Ann Arbor:CFIR Research Team-Center for Clinical Management Research; 2022. [cited 2022 April 12]. Available from: https://cfirguide.org/
15. Damschroder LJ, Lowery JC. Evaluation of a large-scale weight management program using the consolidated framework for implementation research (CFIR). Implement Sci. 2013 May 10;8:51.
16. *Chokotho LC, Mulwafu W, Nyirenda M, Mbomuwa FJ, Pandit HG, Le G, et al. Establishment of trauma registry at Queen Elizabeth Central Hospital (QECH), Blantyre, Malawi and mapping of high risk geographic areas for trauma. World J Emerg Med. 2019;10(1):33-41.*
17. *Croke K, Chokotho L, Milusheva S, Bertfelt J, Karpe S, Mohammed M, et al. Implementation of a multi-center digital trauma registry: Experience in district and central hospitals in Malawi. Int J Health Plann Manage. 2020 Sep;35(5):1157-1172.*
18. *Hamadani F, Razek T, Massinga E, Gupta S, Muataco M, Muripiha P, et al. Trauma surveillance and registry development in Mozambique: results of a 1-year study and the first phase of national implementation. World J Surg. 2019 Jul;43(7):1628-1635.*

For peer review only - http://bmjopen.bmj.com/site/about/guidelines.xhtml

19. *Sawe HR, Sirili N, Weber E, Coats TJ, Reynolds TA, Wallis LA. Perceptions of health providers towards the use of standardised trauma form in managing trauma patients: a qualitative study from Tanzania. Inj Epidemiol. 2020 May 1;7(1):15.*

20. . *Sawe HR, Sirili N, Weber E, Coats TJ, Wallis LA, Reynolds TA. Barriers and facilitators to implementing trauma registries in low-and middle-income countries: qualitative experiences from Tanzania. Afr J Emerg Med. 2020;10(Suppl 1):S23-S28.*

21. *Hashmi ZG, Haider AH, Zafar SN, Kisat M, Moosa A, Siddiqui F, et al. Hospitalbased trauma quality improvement initiatives: first step toward improving trauma outcomes in the developing world. J Trauma Acute Care Surg. 2013 Jul;75(1):60-68.*

22. *Mehmood A, Razzak JA, Kabir S, MacKenzie EJ, Hyder AA. Development and pilot implementation of a locally developed Trauma Registry: lessons learnt in a low-income country. BMC Emerg Med. 2013 Dec 21;13:4.*

23. *Stelfox HT, Joshipura M, Chadbunchachai W, Ellawala RN, O'Reilly G, Nguyen TS, et al. Trauma quality improvement in low and middle income countries of the Asia– Pacific region: a mixed methods study. World J Surg. 2012 Aug;36(8):1978-1992.*

24. *Stevens KA, Paruk F, Bachani AM, Wesson HH, Wekesa JM, Mburu J,et al. Establishing hospital-based trauma registry systems: lessons from Kenya. Injury. 2013 Dec;44 (Suppl 4):S70-S74.*

25. *Fuangworawong P, LaGrone LN, Chadbunchachai W, Sornsrivichai V, Mock CN, Vavilala MS. Assessment of trauma quality improvement activities at public hospitals in Thailand. Int J Surg. 2016 Sep;33 Pt A:88-95.*

26. *Monteverde E, Santero M, Bosque L, Lartigue B, Tenaillon C, Baliña J, et al. A public–private collaborative model for a trauma program implementation: findings from a prospective trauma registry at 14 hospitals in Buenos Aires, Argentina. Eur J Trauma Emerg Surg. 2021 Dec;47(6):1931-1937.*

27. *Parreira JG, Campos TD, Perlingeiro JA, Soldá SC, Assef JC, Gonçalves AC, et al. Implementation of the trauma registry as a tool for quality improvement in trauma care in a Brazilian hospital: the first 12 months. Rev Col Bras Cir. 2015 Jul;42(4):265- 272.*

28. *Schuurman N, Cinnamon J, Matzopoulos R, Fawcett V, Nicol A, Hameed SM. Collecting injury surveillance data in low-and middle-income countries: The Cape Town Trauma Registry pilot. Glob Public Health. 2011;6(8):874-889.*

For peer review only - http://bmjopen.bmj.com/site/about/guidelines.xhtml

29. *Wainiqolo I, Kafoa B, McCaig E, Kool B, McIntyre R, Ameratunga S. Development and piloting of the Fiji injury surveillance in hospitals system (TRIP Project-1). Injury. 2013 Jan;44(1):126-131.*
30. *Zhou J, Wang T, Belenkiy I, Hardcastle TC, Rouby JJ, Jiang B. Management of severe trauma worldwide: implementation of trauma systems in emerging countries: China, Russia and South Africa. Crit Care. 2021 Aug 9;25(1):286.*
31. *Hollinshead L, Jones F, Silvester L, Marshall-Taylor P. Implementing an integrated approach to self-management support in an acute major trauma therapy team: an improvement project. BMJ Open Qual. 2019 Jul 4;8(3):e000415.*
32. *Murphy M, Curtis K, McCloughen A. Facilitators and barriers to the clinical application of teamwork skills taught in multidisciplinary simulated trauma team training. Injury. 2019 May;50(5):1147-1152.*
33. *Newcomb AB, Zadnik M, Carlini AR, Francis MM, Frey KP, Heins SE, et al. Barriers and facilitators to the implementation of injury prevention programs: a qualitative exploration and model development. J Trauma Nurs. 2020;27(6):335-345.*
34. *Wiertsema SH, Donker MH, van Dongen JM, Geleijn E, Bloemers FW, Ostelo RW, et al. The Transmural Trauma Care Model can be implemented well but some barriers 46 and facilitators should be considered during implementation: a mixed methods study. J Physiother. 2021 Oct;67(4):298-307.*
35. *Rosenkrantz L, Schuurman N, Arenas C, Jimenez MF, Hameed MS. Understanding the barriers and facilitators to trauma registry development in resource-constrained settings: a survey of trauma registry stewards and researchers. Injury. 2021 Aug;52(8):2215-2224.*
36. *St-Louis E, Paradis T, Landry T, Poenaru D. Factors contributing to successful trauma registry implementation in low-and middle-income countries: a systematic review. Injury. 2018 Dec;49(12):2100-2110.*
37. *LaGrone LN, Pozo DA, Figueroa JF, Artunduaga MA, Egoavil EH, Castro MJ, et al. Status of trauma quality improvement programs in the Andean region: what foundation do we have to build on. Injury. 2017 Sep;48(9):1985-1993.*
38. *Zargaran E, Schuurman N, Nicol AJ, Matzopoulos R, Cinnamon J, Taulu T, et al. The electronic Trauma Health Record: design and usability of a novel tablet-based tool for

For peer review only - http://bmjopen.bmj.com/site/about/guidelines.xhtml

trauma care and injury surveillance in low resource settings. J Am Coll Surg. 2014 Jan;218(1):41-50.

39. Bommakanti K, Feldhaus I, Motwani G, Dicker RA, Juillard C. Trauma registry implementation in low-and middle-income countries: challenges and opportunities. J Surg Res. 2018 Mar;223:72-86.*
40. *O'Reilly GM, Gabbe B, Braaf S, Cameron PA. An interview of trauma registry custodians to determine lessons learnt. Injury. 2016 Jan;47(1):116-1
41. *Wild H, Mock C, Lim A. Implementation of the WHO Trauma Care Checklist: A qualitative analysis of facilitators and barriers to use. Int J Surg. 2020 Nov;83:15-23.*
42. *Rosenkrantz L, Schuurman N, Arenas C, Nicol A, Hameed MS. Maximizing the potential of trauma registries in low-income and middle-income countries. Trauma Surg Acute Care Open. 2020 May 12;5(1):e000469.*
43. Parker LE, Kirchner JE, Bonner LM, Fickel JJ, Ritchie MJ, Simons CE, et al. Creating a quality-improvement dialogue: utilising knowledge from frontline staff, managers, and experts to foster health care quality improvement. Qual Health Res. 2009 Feb;19(2):229-242
44. Santana MJ, Straus S, Gruen R, Stelfox HT. A qualitative study to identify opportunities for improving trauma quality improvement. J Crit Care. 2012 Dec;27(6):738.e1-7.
45. Rosenkrantz L, Schuurman N, Hameed M. Trauma registry implementation and operation in low and middle income countries: a scoping review. Glob Public Health. 2019 Dec;14(12):1884-1897.
46. O'Reilly GM, Gabbe B, Cameron PA. Trauma registry methodology: a survey of trauma registry custodians to determine current approaches. Injury. 2015 Feb;46(2):201-206.
47. Sifrim ZK, Barker PM, Mate KS. What gets published: the characteristics of quality improvement research articles from low-and middle-income countries. BMJ Qual Saf. 2012 May;21(5):423-431
48. Keller PR, Schneeberger S, Drolet BC, Al Kassis S, Pearce FB, Perdikis G. Implementation of a trauma quality improvement program communications package decreases time-to-operation for facial trauma. Ann Plast Surg. 2019 Jun;82(6S Suppl 5):S380-S385.

For peer review only - http://bmjopen.bmj.com/site/about/guidelines.xhtml

49. Bardfield J, Agins B, Akiyama M, Basenero A, Luphala P, Kaindjee-Tjituka F, et al. A quality improvement approach to capacity building in low-and middle-income countries. Aids. 2015 Jul;29(Suppl 2):S179-S186.
50. O'Reilly GM, Gabbe B, Moore L, Cameron PA. Classifying, measuring and improving the quality of data in trauma registries: a review of the literature. Injury. 2016 Mar;47(3):559-567.
51. Porgo TV, Moore L, Tardif PA. Evidence of data quality in trauma registries: a systematic review. J Trauma Acute Care Surg. 2016 Apr;80(4):648-658.
52. Whitaker J, Nepogodiev D, Leather A, Davies J. Assessing barriers to quality trauma care in low and middle-income countries: a Delphi study. Injury. 2020 Feb;51(2):278- 285
53. Gosselin RA, Spiegel DA, Coughlin R, Zirkle LG. Injuries: the neglected burden in developing countries. Bull World Health Organ. 2009 Apr;87(4):246-246a.
54. Arksey H, O'Malley L. Scoping studies: towards a methodological framework. Int J Soc Res Methodol. 2005 Feb;8(1):19-32.

Note: * studies included in the review*

Acknowledgements:

We would like to acknowledge Karolinska Institutet Library (Independent Information Specialist Consultants, Solna, Sweden) for peer review of the literature search strategy, along with the faculty and students of Karolinska Institutet Department of Global Public Health for comments and feedback on earlier versions of this manuscript.

Funding

No funding sources used

Availability of data and materials

The data that support the findings of this study are available from PubMed, Web of Science, and Global Index Medicus databases using the search string outlined in Online Supplement 1.

For peer review only - http://bmjopen.bmj.com/site/about/guidelines.xhtml

Authors' contributions

Study concept, design and search strategy: GK, JB, MGW. Data selection and screening: GK, JB, YS, MGW. Data analysis and interpretation: GK, JB, MGW. Drafting of manuscript: GK, JB, MGW. Critical revisions of manuscript: GK, JB, YS, MGW. Study supervision: JB, MGW. All authors read and approved the final manuscript.

Ethics Declarations:

Ethics approval and consent to participate

Not applicable

Consent for publication

Not applicable

Competing interests

The authors declare that they have no competing interests

For peer review only - http://bmjopen.bmj.com/site/about/guidelines.xhtml

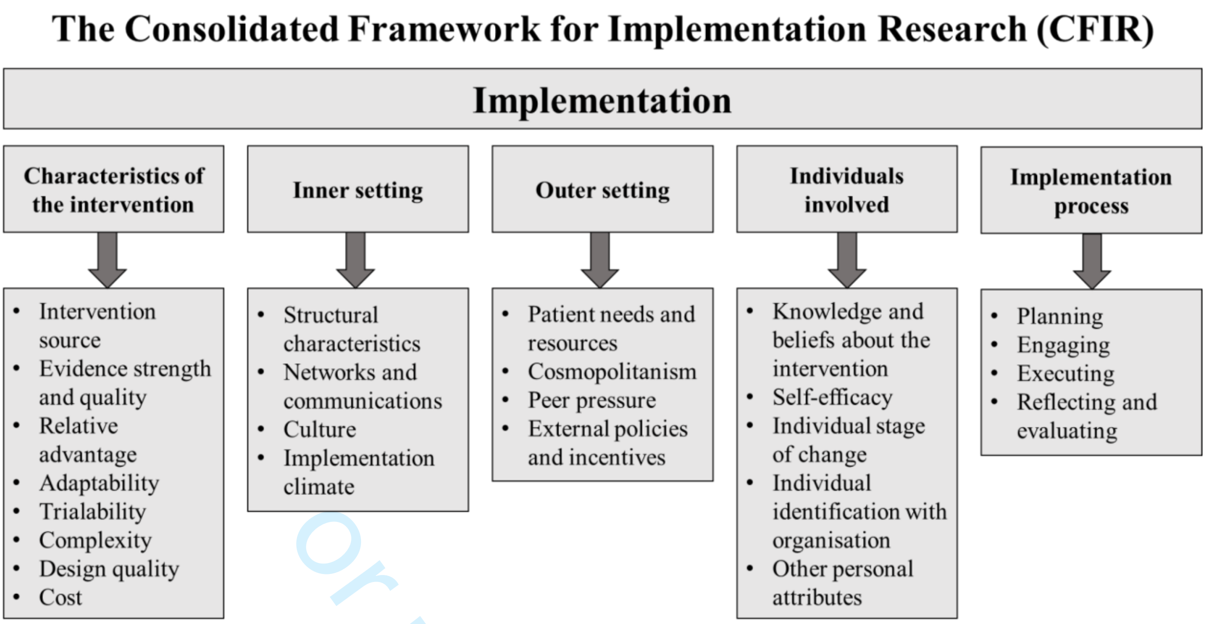

For peer review only

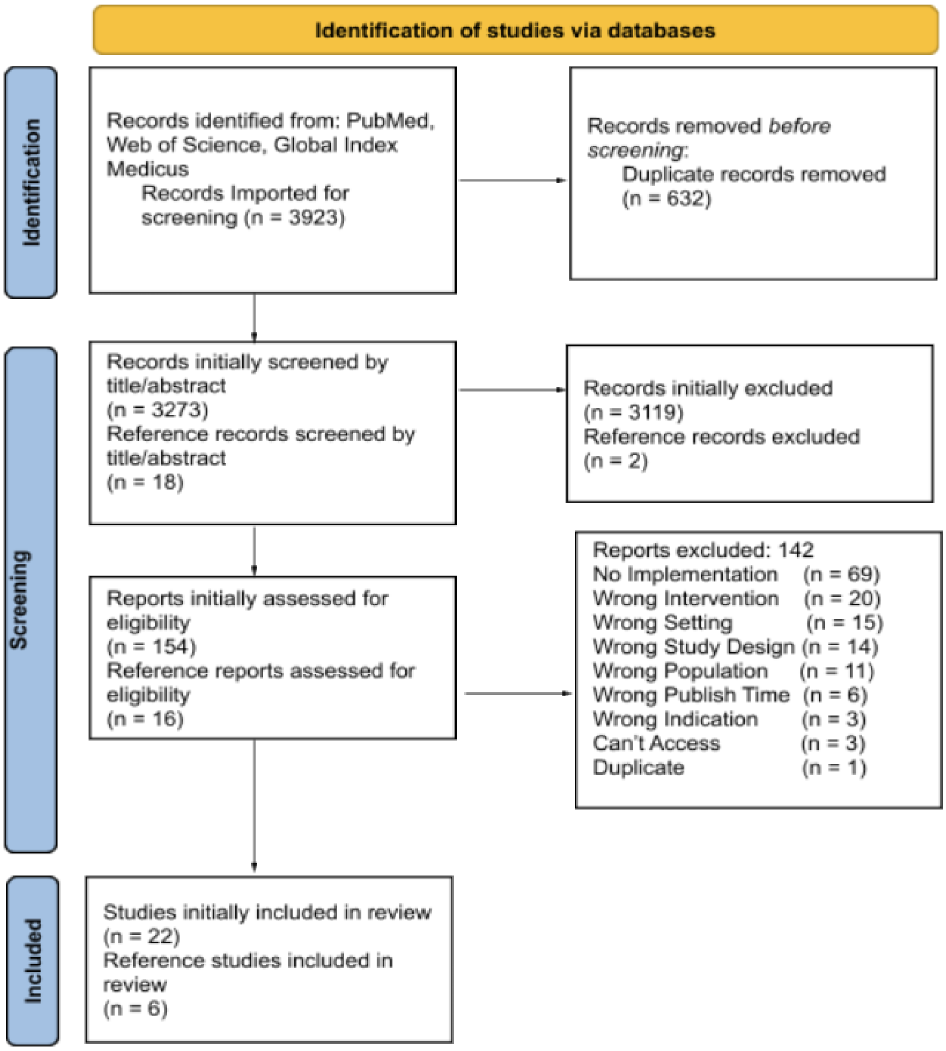
Identification of studies via databases
Identification
Records identified from: PubMed, Web of Science, Global Index Medicus
Records Imported for screening (n = 3923)
Records removed *before screening*:
Duplicate records removed (n = 632)
Screening
Records initially screened by title/abstract (n = 3273)
Reference records screened by title/abstract (n = 18)
Records initially excluded (n = 3119)
Reference records excluded (n = 2)
Reports initially assessed for eligibility (n = 154)
Reference reports assessed for eligibility (n = 16)
Reports excluded: 142
No Implementation (n = 69)
Wrong Intervention (n = 20)
Wrong Setting (n = 15)
Wrong Study Design (n = 14)
Wrong Population (n = 11)
Wrong Publish Time (n = 6)
Wrong Indication (n = 3)
Can't Access (n = 3)
Duplicate (n = 1)
Included
Studies initially included in review (n = 22)
Reference studies included in review (n = 6)

Online Supplement:

Online Supplement 1: Database search strategy according to Population, Concept, Context (PCC) framework

**Population:**		
**#1**	**MeSH terms:**	Humans and Trauma Centres
**#2**	**Free Text:**	Population OR populations OR patient OR patients OR adult OR adults OR injured OR victim OR victims OR trauma centre OR trauma centres OR trauma department OR trauma departments OR trauma facility OR trauma facilities OR acute care centre OR acute care centres OR acute care department OR acute care departments OR acute care facility OR acute care facilities OR primary care centre OR primary care centres OR primary care department OR primary care departments OR primary care facility OR primary facilities OR emergency centre OR emergency centres OR emergency department OR emergency departments OR emergency facility OR emergency facilities
**#3**	**#1 OR #2**	
**Concept:**		
**#4**	**MeSH terms:**	Hospital and quality improvement
**#5**	**Free Text:**	Hospital OR hospitals OR in-hospital OR hospital-based OR facility OR facilities AND quality improvement OR quality indicator OR facilitator OR facilitators OR opportunity OR opportunities OR advantage OR advantages OR solution OR solutions OR barrier OR barriers OR challenge OR challenges OR disadvantage OR disadvantages OR inhibitor OR inhibitors OR implementation OR intervention OR innovation
**#6**	**#4 OR #5**	
**Context:**		
**#7**	**MeSH terms:**	Developing countries and developed countries
**#8**	**Free Text:**	Developing country OR developing countries OR developing nation OR developing nations OR developing economy OR developing economies OR low income country OR low income countries OR low income nation OR low income nations OR low income economy OR low income economies OR lower middle income

		country OR lower middle income countries OR lower middle income nation OR lower middle income nations OR lower middle income economy OR lower middle income economies OR upper middle income country OR upper middle income countries OR upper middle income nation OR upper middle income nations OR upper middle income economy OR upper middle income economies OR middle income country OR middle income countries OR middle income nation OR middle income nations OR middle income economy OR middle income economies OR transitional country OR transitional countries OR transitional nation OR transitional nations OR transitional economy OR transitional economies OR developed country OR developed countries OR developed nation OR developed nations OR developed economy OR developed economies OR high income country OR high income countries OR high income nation OR high income nations OR high income economy OR high income economies
**#9**	**#7 OR #8**	
**#10**	**#3 AND #6 OR #9**	3923 Results
**Filter**	English language studies published from June 2009 onwards	

Online Supplement 2: Characteristics of extracted studies

Income Level	Continents/Countries	Source	Study Type	Intervention Type	Intervention Duration (months)
L	Malawi	Chokotho et al. 2019 [16]	Qualitative - Survey	T.R	N/A
L	Malawi	Croke et al. 2020 [17]	Qualitative - Survey	T.R	N/A
L	Mozambique	Hamadani et al. 2019 [18]	Qualitative - Interview	T.R	12
L	Tanzania[a]	Sawe et al. 2020 [19]	Qualitative - Focus Group Discussion	T.R	N/A
L	Tanzania[a]	Sawe et al. 2020 [20]	Qualitative - Interview	T.R	N/A
LM	Pakistan	Hashmi et al. 2013 [21]	Quantitative - logistic regression	T.R, Q.A.P	108
LM	Pakistan	Mehmood et al. 2013 [22]	Qualitative - Survey	T.R	3

For peer review only

LM	Asia: 7	Stelfox et al. 2012 [23]	Mixed Methods	Range - M&M, PDP, AF, QOC Audit, Statistical Analysis	N/A
LM	Kenya	Stevens et al. [24]	Qualitative - Survey	T.R	N/A
UM	Thailand	Fuangworawong et al. 2016 [25]	Qualitative - Survey	Range - M&M, T.R, A.F, PDP	N/A
UM	Peru	LaGrone et al. 2017 [10]	Mixed methods	Range - M&M/T.R	N/A
UM	Argentina	Monteverde et al. 2021 [26]	Qualitative - Univariate/Bivariate descriptive analysis	T.R	114
UM	Brazil	Parreira et al. 2015 [27]	Qualitative - Survey	T.R	12
UM	South Africa	Schuurman et al. 2011 [28]	Qualitative - Survey	T.R	1
UM	Fiji	Wainiqolo et al. 2013 [29]	Qualitative - Survey	T.R	5
UM	Africa: 1, Asia: 1, Europe: 1	Zhou et al. 2021 [30]	Literature Review	T.R	N/A
H	U.K	Hollinshead et al. 2019 [31]	Qualitative - Survey	Educational - B.S.M.P	12
H	Australia	Murphy et al. 2019 [32]	Qualitative - Survey	Multidisciplinary TTT	N/A
H	U.S.A	Newcomb et al. 2020 [33]	Qualitative - Interview	I.P.P	N/A
H	Netherlands	Wiertsema et al. 2021 [34]	Mixed Methods	TTCM	N/A
Range - LMIC	Africa: 6, Asia: 3, South America: 3, North America: 1	Rosenkrantz et al. 2021 [35]	Qualitative - Survey	T.R	Range - 3 = <24 1 = 24-60, 7 = 72-120, 2 = >120
Range - LMIC	Africa: 9, Asia: 4, South	St-Louis et al. 2018 [36]	Literature Review	T.R	N/A

	America: 3, North America: 1, Oceania: 1				
Range - LM/UM	South America: 4	LaGrone et al. 2017 [37]	Qualitative - Survey	Range - M&M, T.R, A.F, Autopsies	N/A
Range - UM/H	South Africa/Canada	Zargaran et al. 2014 [38]	Qualitative - Survey	T.R	1
Range - All	Africa: 6, Asia: 6, North America: 3, South America: 2, Europe: 1, Oceania: 1	Bommakanti et al. 2018 [39]	Literature Review	T.R	N/A
Range - All	Africa: 8, Asia: 6, Europe: 3, North America: 3, Oceania: 2	O'Reilly et al. 2016 [40]	Qualitative - Interview	T.R	N/A
Range - All	Asia: 5, Africa: 3, South America: 1, North America: 1, Oceania: 1	Wild et al. 2020 [41]	Qualitative - Interview	TCC	24
Range - Unspecified	Africa: 7, Asia: 3, North America: 2, Europe: 1, Oceania: 1	Rosenkrantz et al. 2020 [42]	Literature Review	T.R	N/A

Note. L = Low Income, LM = Lower Middle Income, UM = Upper Middle Income, H = High Income, T.R = Trauma Registry, Q.A.P = Quality Assurance Program, M&M = Mortality & Morbidity Conferences, PDP = Preventable Death Panel, A.F = Audit Filter, QOC = Quality of Care, B.S.M.P = Bridges Self-Management Programme, TTT = Trauma Team Training, I.P.P = Injury Prevention Program, TTCM = Transmural Trauma Care Model, TCC = Trauma Care Checklist.

"Qualitative - Survey" design included studies that conducted questionnaires

[a]Tanzania reclassified from low to lower middle income country after data collection of studies

For peer review only

Preferred Reporting Items for Systematic reviews and Meta-Analyses extension for Scoping Reviews (PRISMA-ScR) Checklist

SECTION	ITEM	PRISMA-ScR CHECKLIST ITEM	REPORTED ON PAGE #
**TITLE**			
Title	1	Identify the report as a scoping review.	1
**ABSTRACT**			
Structured summary	2	Provide a structured summary that includes (as applicable): background, objectives, eligibility criteria, sources of evidence, charting methods, results, and conclusions that relate to the review questions and objectives.	2
**INTRODUCTION**			
Rationale	3	Describe the rationale for the review in the context of what is already known. Explain why the review questions/objectives lend themselves to a scoping review approach.	4
Objectives	4	Provide an explicit statement of the questions and objectives being addressed with reference to their key elements (e.g., population or participants, concepts, and context) or other relevant key elements used to conceptualize the review questions and/or objectives.	4
**METHODS**			
Protocol and registration	5	Indicate whether a review protocol exists; state if and where it can be accessed (e.g., a Web address); and if available, provide registration information, including the registration number.	5
Eligibility criteria	6	Specify characteristics of the sources of evidence used as eligibility criteria (e.g., years considered, language, and publication status), and provide a rationale.	5
Information sources*	7	Describe all information sources in the search (e.g., databases with dates of coverage and contact with authors to identify additional sources), as well as the date the most recent search was executed.	5
Search	8	Present the full electronic search strategy for at least 1 database, including any limits used, such that it could be repeated.	Online Supplement 1
Selection of sources of evidence†	9	State the process for selecting sources of evidence (i.e., screening and eligibility) included in the scoping review.	5
Data charting process‡	10	Describe the methods of charting data from the included sources of evidence (e.g., calibrated forms or forms that have been tested by the team before their use, and whether data charting was done independently or in duplicate) and any processes for obtaining and confirming data from investigators.	5
Data items	11	List and define all variables for which data were sought and any assumptions and simplifications made.	5
Critical appraisal of individual sources of evidence§	12	If done, provide a rationale for conducting a critical appraisal of included sources of evidence; describe the methods used and how this information was used in any data synthesis (if appropriate).	N/A

St. Michael's
Inspired Care.
Inspiring Science.

For peer review only - http://bmjopen.bmj.com/site/about/guidelines.xhtml

SECTION	ITEM	PRISMA-ScR CHECKLIST ITEM	REPORTED ON PAGE #
Synthesis of results	13	Describe the methods of handling and summarizing the data that were charted.	6
**RESULTS**			
Selection of sources of evidence	14	Give numbers of sources of evidence screened, assessed for eligibility, and included in the review, with reasons for exclusions at each stage, ideally using a flow diagram.	7
Characteristics of sources of evidence	15	For each source of evidence, present characteristics for which data were charted and provide the citations.	Online Supplement 2
Critical appraisal within sources of evidence	16	If done, present data on critical appraisal of included sources of evidence (see item 12).	N/A
Results of individual sources of evidence	17	For each included source of evidence, present the relevant data that were charted that relate to the review questions and objectives.	7-15
Synthesis of results	18	Summarize and/or present the charting results as they relate to the review questions and objectives.	15
**DISCUSSION**			
Summary of evidence	19	Summarize the main results (including an overview of concepts, themes, and types of evidence available), link to the review questions and objectives, and consider the relevance to key groups.	16-18
Limitations	20	Discuss the limitations of the scoping review process.	18
Conclusions	21	Provide a general interpretation of the results with respect to the review questions and objectives, as well as potential implications and/or next steps.	18-19
**FUNDING**			
Funding	22	Describe sources of funding for the included sources of evidence, as well as sources of funding for the scoping review. Describe the role of the funders of the scoping review.	25

JBI = Joanna Briggs Institute; PRISMA-ScR = Preferred Reporting Items for Systematic reviews and Meta-Analyses extension for Scoping Reviews.

* Where *sources of evidence* (see second footnote) are compiled from, such as bibliographic databases, social media platforms, and Web sites.

† A more inclusive/heterogeneous term used to account for the different types of evidence or data sources (e.g., quantitative and/or qualitative research, expert opinion, and policy documents) that may be eligible in a scoping review as opposed to only studies. This is not to be confused with *information sources* (see first footnote).

‡ The frameworks by Arksey and O'Malley (6) and Levac and colleagues (7) and the JBI guidance (4, 5) refer to the process of data extraction in a scoping review as data charting.

§ The process of systematically examining research evidence to assess its validity, results, and relevance before using it to inform a decision. This term is used for items 12 and 19 instead of "risk of bias" (which is more applicable to systematic reviews of interventions) to include and acknowledge the various sources of evidence that may be used in a scoping review (e.g., quantitative and/or qualitative research, expert opinion, and policy document).

From: Tricco AC, Lillie E, Zarin W, O'Brien KK, Colquhoun H, Levac D, et al. PRISMA Extension for Scoping Reviews (PRISMAScR): Checklist and Explanation. Ann Intern Med. 2018;169:467–473. doi: 10.7326/M18-0850.

St. Michael's
Inspired Care. Inspiring Science.

For peer review only - http://bmjopen.bmj.com/site/about/guidelines.xhtml

BMJ Open

Facilitators and barriers impacting in-hospital Trauma Quality Improvement Program (TQIP) implementation across country income levels: A scoping review

Journal:	*BMJ Open*
Manuscript ID	bmjopen-2022-068219.R1
Article Type:	Original research
Date Submitted by the Author:	17-Jan-2023
Complete List of Authors:	Kapanadze, George; Karolinska Institute, Global Public Health<br>Berg, Johanna; Karolinska Institute, Global Public Health; Skånes universitetssjukhus Malmö, Emergency and Internal medicine<br>Sun, Yue; Karolinska Institute, Global Public Health<br>Gerdin Wärnberg, Martin ; Karolinska Institutet, Department of Global Public Health; Karolinska University Hospital, Function Perioperative Medicine and Intensive Care
**Primary Subject Heading**:	Global health
Secondary Subject Heading:	Public health, Qualitative research
Keywords:	Quality in health care < HEALTH SERVICES ADMINISTRATION & MANAGEMENT, TRAUMA MANAGEMENT, Change management < HEALTH SERVICES ADMINISTRATION & MANAGEMENT

SCHOLARONE™
Manuscripts

I, the Submitting Author has the right to grant and does grant on behalf of all authors of the Work (as defined in the below author licence), an exclusive licence and/or a non-exclusive licence for contributions from authors who are: i) UK Crown employees; ii) where BMJ has agreed a CC-BY licence shall apply, and/or iii) in accordance with the terms applicable for US Federal Government officers or employees acting as part of their official duties; on a worldwide, perpetual, irrevocable, royalty-free basis to BMJ Publishing Group Ltd ("BMJ") its licensees and where the relevant Journal is co-owned by BMJ to the co-owners of the Journal, to publish the Work in this journal and any other BMJ products and to exploit all rights, as set out in our licence.

The Submitting Author accepts and understands that any supply made under these terms is made by BMJ to the Submitting Author unless you are acting as an employee on behalf of your employer or a postgraduate student of an affiliated institution which is paying any applicable article publishing charge ("APC") for Open Access articles. Where the Submitting Author wishes to make the Work available on an Open Access basis (and intends to pay the relevant APC), the terms of reuse of such Open Access shall be governed by a Creative Commons licence – details of these licences and which Creative Commons licence will apply to this Work are set out in our licence referred to above.

Other than as permitted in any relevant BMJ Author's Self Archiving Policies, I confirm this Work has not been accepted for publication elsewhere, is not being considered for publication elsewhere and does not duplicate material already published. I confirm all authors consent to publication of this Work and authorise the granting of this licence.

For peer review only

Facilitators and barriers impacting in-hospital Trauma Quality Improvement Program (TQIP) implementation across country income levels: A scoping review

George Kapanadze, MMSc; Johanna Berg, MD; Yue Sun, MPH; Martin Gerdin Wärnberg MD, PhD

Author Affiliations: Department of Global Public Health, Karolinska Institutet, Solna, Sweden (Kapanadze); Department of Global Public Health, Karolinska Institutet, Solna, Sweden, Department of Internal and Emergency Medicine, Skåne University Hospital , Malmö (Berg); Department of Global Public Health, Karolinska Institutet, Solna, Sweden (Sun); Department of Global Public Health, , Karolinska Institutet, Solna, Sweden; Perioperative Medicine and Intensive Care, Karolinska University Hospital, Solna, Sweden (Gerdin Wärnberg).

Corresponding Author: George Kapanadze, MMSc, Department of Global Public Health, Karolinska Institutet,Widerströmska huset Tomtebodavägen 18 A, plan 3, 171 65 Solna, Sweden (george.kapanadze@stud.ki.se)

For peer review only

Abstract:

Objective: Trauma is a leading cause of mortality and morbidity globally, disproportionately affecting low and middle income countries (LMICs). Understanding the factors determining implementation success for in-hospital Trauma Quality Improvement Programs (TQIPs) is critical to reducing the global trauma burden. We synthesised topical literature to identify key facilitators and barriers to in-hospital TQIP implementation across country income levels.

Design: Scoping review using the Preferred Reporting Items for Systematic Reviews and Meta-Analyses extension (PRISMA-ScR)

Data Sources: PubMed, Web of Science, and Global Index Medicus databases were searched from June 2009 – January 2022.

Eligibility criteria for selecting studies: Published literature involving any study design, written in English, and evaluating any implemented in-hospital quality improvement program in trauma populations worldwide. Literature that was non-English, unpublished and involved non-hospital TQIPs was excluded.

Data extraction and synthesis: Two reviewers completed a three-stage screening process using Covidence, with any discrepancies resolved through a third reviewer. Content analysis using the Consolidated Framework for Implementation Research (CFIR) identified facilitator and barrier themes for in-hospital TQIP implementation.

Results: Twenty-eight studies met the eligibility criteria from 3923 studies. The most discussed in-hospital TQIP in included literature were trauma registries. Facilitators and barriers were similar across all country income levels. The main facilitator themes identified were the prioritisation of staff education and training, strengthening stakeholder dialogue, and providing standardised best-practice guidelines. The key barrier theme identified in LMICs was poor data quality while high-income countries (HICs) had reduced communication across professional hierarchies.

For peer review only

Conclusions: Stakeholder prioritisation of in-hospital TQIPs, along with increased knowledge and consensus of trauma care best practices are essential efforts to reduce the global trauma burden. The primary focus of future studies on in-hospital TQIPs in LMICs should target improving registry data quality, while interventions in HICs should target strengthening communication channels between healthcare professionals.

Strengths and limitations of this study:

- The present scoping review was based on a comprehensive search of published literature that included a robust screening process involving multiple peer reviewers to protect against bias.
- We used the Consolidated Framework for Implementation Research (CFIR) to ground collected evidence in an established theory.
- The generalisability of our results was impacted by grouping literature based on country income level at the time of publication as we did not investigate potential differences in available resources within countries (e.g., regions and healthcare institutions).
- The lack of quality assessment involved in scoping review design limits the implications of gathered results, given the focus on mapping available literature, and identifying knowledge gaps.

For peer review only

Introduction:

Trauma is the clinical entity composed of physical injury and the body's associated response [1]. Trauma is a leading cause of death and disability worldwide [2]. To improve in-hospital care, multiple Trauma Quality Improvement Programs (TQIPs) have been developed, typically originating in high-income countries (HICs). Key in-hospital TQIPs highlighted by the World Health Organisation (WHO) in their 2009 guidelines included techniques such as trauma registries along with mortality and morbidity conferences (3). The implementation of TQIPs have been attributed to significant improvements in care quality and outcomes in HICs [4].

Quality Improvement is designed to achieve positive change in a specific process [5], which can be highly dependent on the perspectives, attitudes, and behaviours of patients and healthcare professionals in their local context [6]. Although quality improvement aims to improve the quality of care, organisations often struggle with its implementation [7]. While implementation research has improved significantly, there remain substantial gaps in knowledge and understanding of factors that facilitate or impede efforts to implement quality improvement in healthcare organisations [8].

Despite research on developing in-hospital TQIPs steadily rising, little context-relevant guidance exists to help policymakers set priorities for implementation [9]. A recent study found a lack of in-depth examination of barriers and facilitators to TQIP implementation in low and middle income countries (LMIC) [10]. This exemplifies the need for further research to assess trauma systems in LMICs to inform health system strengthening for trauma care [4]. Thus, the scoping review aimed to identify key facilitators and barriers to in-hospital TQIP implementation across country income levels.

For peer review only - http://bmjopen.bmj.com/site/about/guidelines.xhtml

Methods:

We conducted a scoping review to understand available evidence on factors influencing in-hospital TQIP implementation in accordance with the Preferred Reporting Items for Systematic Reviews and Meta-Analyses extension for Scoping Reviews (PRISMA-ScR) [11]. A scoping review was most appropriate due to the broad nature of the topic along with the wide variety of potential factors impacting quality improvement implementation in differing income settings. We defined country income level as the income level of each included country based on World Bank classification at the time of publication [12].

Search Strategy

The search strategy was developed with a librarian using keywords, synonyms, and medical subject headings (MeSH) as shown in online supplement 1**.** The review searched three databases, *PubMed*, *Web of Science* and *Global Index Medicus*, for studies from June 2009 to January 2022. The search was limited to publications from June 2009 onwards in line with the WHO release on Guidelines for TQIPs (3).

Study Selection

Given the review's broad scope, all in-hospital TQIPs were considered, along with studies of any design. The screening process was completed by two authors, assisted by a third reviewer. The following inclusion criteria were used:

Any English language studies evaluating facilitators and barriers to implementation of system-wide in-hospital quality improvement programs in trauma populations worldwide.

Studies were excluded if they were:

Non-English, unpublished, if they described projects where implementation was not a focus, or if they discussed non-hospital TQIPs.

Data Charting

Data extraction was performed using a predetermined form addressing study identifying information; geographic location; population and study setting; country income level at the time of publication; data collection methods; quality improvement intervention type and duration;

For peer review only - http://bmjopen.bmj.com/site/about/guidelines.xhtml

implementation facilitators and barriers; implementation outcome and study funding sources. Appropriate quotations were identified and extracted through deductive content analysis. We did not evaluate the evidence underlying the identified TQIPs, as our focus was on facilitators and barriers to implementing a TQIP once attempted.

Summarising and Reporting Results

Facilitator and barrier selection was guided by the Consolidated Framework for Implementation Research (CFIR) - illustrated in figure 1. The framework is composed of five domains: intervention characteristics, outer setting, inner setting, individual characteristics, and implementation process [13]. These domains provide a practical guide for systematically assessing potential facilitators and barriers to TQIP implementation [14], while offering insights to explain differences across income levels [15].

Patient and Public Involvement

Patients and the public were not involved in the design and conduct of this research.

For peer review only

Results:

The literature search yielded 3923 studies, 3291 studies passed title and abstract screening while 170 reports were identified for full-text screening. The number of extracted studies was 28, with details on study selection found in figure 2. Most of the studies originated in upper middle income countries (7 [25%]), followed by low income (5 [18%]), high income (4 [14%]), and lower middle income (4 [14%]). The remaining studies (8 [29%]) included multi-country analyses that spanned across income levels. The most represented continents in the review were Africa and Asia, with South Africa (11 [39%]) and Pakistan (9 [32%]) appearing most frequently. Most studies solely discussed trauma registries as the prominent in-hospital TQIP (18 [64%]) while a small number evaluated two or more TQIPs in their reports (5 [18%]). Full study characteristic details are found in online supplement 2.

The CFIR domains identified most in the literature were intervention characteristics and inner setting. A summary of facilitators and barriers according to the CFIR are in Tables 1 and 2.

Table 1: Summary of in-hospital TQIP implementation facilitators using the CFIR

CFIR Domain	CFIR Construct	Facilitators	Quotes
Intervention Characteristics	Relative Advantage	Open dialogue with key stakeholders	1. *Active cooperation and extensive input from Ministry of Health stakeholders* [16]<br>2. *Appealing to stakeholders higher up in the Department of Health* [17]
	Adaptability	Tailoring intervention to local context	1. *All data collection was carried out by local Mozambican health workers* [18]<br>2. *Participants highlighted that patient care should be adapted to local circumstances* [19]
	Complexity	Focus on simplicity	1. *Balance between sufficient detail and simplicity of the data collection process* [20]<br>2. *Less is more when it comes to trauma registry* [21]

For peer review only - http://bmjopen.bmj.com/site/about/guidelines.xhtml

Outer Setting	Patient needs & Resources	Prioritising patient care	1. *More focus on the everyday things that patients have to deal with* [22]<br>2. *Harnessing…clinical enthusiasm to improve trauma care* [21]
	External policies & incentives	Collaboration with national/international bodies	1. *Increased focus on trauma academics through regional and international collaborations* [23]<br>2. *Seek potential collaborators with interest* [17]
Inner Setting	Implementation Climate		
	Learning Climate	Staff education & training	1. *Engage nursing and medical staff in multidisciplinary training* [24]<br>2. *Training needs to include strategies that address fundamental interpersonal communication* [25]
	Readiness for implementation		
	Leadership engagement	Prioritise buy-in	1. *Leadership was identified as having a clear impact on team factors* [25]<br>2. *The injury prevention coordinators' work was influenced by the support from direct managers and senior leadership* [26]
	Available Resources	Diversify resource sources	1. *External training resources, such as national or state conferences, were important to allow injury prevention champions to connect* [26]<br>2. *Broadening the scope of the registry to apply for additional grants* [17]
Characteristics of Individuals	Knowledge & Beliefs about the intervention	Generate understanding of quality improvement	1. *There is a need for increased dissemination of QI programs as a means of empowering local providers to participate in their health systems* [27]<br>2. *Formulate very clearly a vision, mission and strategy that everyone understands* [21]

For peer review only - http://bmjopen.bmj.com/site/about/guidelines.xhtml

	Other Personal Attributes	Communication training	1. *Important to find ways to sustain changes in clinician communication style* [24]<br>2. *Training needs to include strategies that address fundamental interpersonal communication alongside other solutions* [25]
Process of Implementation	Reflecting & Evaluating	Strengthening data quality	1. *Increasing real and perceived efficacy of mortality & morbidity conferences…through the establishment of standardised case selection criteria* [27]<br>2. *Regular audits and staged validation of data* [21]
	Engaging		
	Champions	Identifying dedicated staff	1. *Sometimes I just get inspired by something that occurs to me as nothing is being done about it currently* [26]<br>2. *Identify a person with passion and zeal to drive the effort* [17]

Table 2: Summary of in-hospital TQIP implementation barriers using the CFIR

CFIR Domain	CFIR Construct	Barriers	Quotes
Intervention Characteristics	Relative Advantage	Limited desire for quality improvement initiatives	1. *A "so what" attitude amongst stakeholders from other nearby facilities* [17]<br>2. *Current lack of motivation for quality improvement programs in Latin America* [27]
	Adaptability	Resistance to change	1. *Surgeon's preference for autonomy and self-reliance over standardisation: "I do it my way, you do it yours"* [10]<br>2. *Allied health professionals identified the barriers of…'the way things are always done"* [24]

For peer review only

	Complexity	Competing interests between groups	1. *Institutional and national prioritisation of other patient care objectives* [10]<br>2. *Trauma is a very low priority for most clinicians* [21]
Outer Setting	Patient needs & Resources	Staff time constraints	1. *Staff have multiple demands on their time* [16]<br>2. *Surgeon spends less time with a patient, he can proceed with the next patient* [22]
	External policies & incentives	Lack of common guidelines	1. *Absence of uniform policies, guidelines, and protocols* [19]<br>2. *No standards or best practices in place for the collection of data* [28]
Inner Setting	Implementation Climate		
	Learning Climate	Professional hierarchies	1. *Senior staff attempting to maintain the status quo in order to fly under the radar* [10]<br>2. *Limited ability to engage leaders* [19]
	Readiness for implementation		
	Leadership engagement	Lack of buy-in	1. *Some stakeholders…created barriers by prioritising other clinical issues* [17]<br>2. *Rest of the stakeholders and the doctors did not buy-in to this concept of using a registry* [21]
	Available Resources	Limited Resources	1. *Hospitals cannot afford to hire specialised personnel solely to collect data* [29]<br>2. *Make do with the resources that you have, because the funding will not be there* [21]
Characteristics of Individuals	Knowledge & Beliefs about the intervention	Lack of staff belief in quality improvement interventions	1. *Some initial skepticism from counterparts* [16]<br>2. *It's not fitting into anybody's career plans at this point in time…trauma happens to poor people* [30]

For peer review only - http://bmjopen.bmj.com/site/about/guidelines.xhtml

	Other Personal Attributes	Lack of staff knowledge	1. *Almost half of newly-graduated physicians do not have experience working with this QI tool during their training* [31]<br>2. *Not a healthy understanding of how we do our work or what's needed to do our work* [26]
Process of Implementation	Reflecting & Evaluating	Poor Data Quality	1. *Challenges reported included the high rates of missing data* [18]<br>2. *A major hurdle is data quality of clinical records* [32]
	Engaging		
	Champions	Overworked Staff	1. *Human resources to meet volume of data collection were underestimated* [19]<br>2. *Constant suggesting by the faculty to use overworked residents to collect trauma data* [17]

Domain 1: Characteristics of the Intervention

Open dialogue with key stakeholders facilitates the implementation process [10,16,17,33]. A multi-centre digital trauma registry implementation in Malawi revealed that consistent communication between Ministry of Health stakeholders and the research team contributed to the success of the registry [16]. Competing interests between groups acted as a barrier to establishing communication channels between stakeholders [10,17,21]. The absence of a vision for trauma care was highlighted by a custodian from a multi-hospital in a developing country as there was a need to *"show the vision of better trauma care to lots of people, because trauma is a very low priority for most clinicians, and it happens to poor people"* [21 p.119].

Another key theme was the importance of adaptability. A focus on tailoring the intervention to the local context to ensure success and sustainability was apparent [10,17,18,19,25,27,34,35]. The implementation of a multi-hospital injury surveillance program in Mozambique was driven by

For peer review only - http://bmjopen.bmj.com/site/about/guidelines.xhtml

local data collectors that enhanced the sustainability of the project [18]. Tailoring programs to local conditions is often made difficult by individuals' resistance to change [10,31]. An educational self-management intervention in a trauma centre in the UK revealed that the roll-out of support was hindered by team members preferring to continue with established practices, which limited widespread adoption [24].

Attention to simplicity is an implementation facilitator [17,36,20,21]. A trauma registry development study in LMICs emphasised the significance of keeping design simple through the standardisation of variables to enhance accuracy and understanding [17]. Standardisation requires widespread staff and stakeholder support, which is often lacking due to a limited desire for quality improvement. A survey of trauma care providers in the Andean region revealed that less than half of respondents *"ever witnessed a change occur in their institution",* which influenced the *"current lack of motivation for quality improvement programs"* [27 p.1992].

Domain 2: Outer Setting

Prioritising patient care during implementation was widely addressed [22,23,24,35]. A Dutch evaluation of the implementation of the Transmural Trauma Care Model found that patients' experience improved as more focus was placed on day-to-day issues patients encountered [22]. However, time constraints were a key barrier to providing improved patient care [10,16,22,24, 31,32,34,37,]. A Peruvian study on the status of in-hospital TQIPs revealed the *"prevalence of dual practice"* [10 p.966], surgeons working at both public and private institutions, meant that clinicians did not have time to effectively participate in emerging quality improvement initiatives.

Collaboration with national and international bodies was a facilitator to successful implementation [17,23]. A questionnaire with trauma registry stewards in LMICs showed a need to *"develop skills at political lobbying to induce support from the administration*" to provide a platform for long-term aid [17 p.2221]. The lack of common guidelines was highlighted as a barrier [10,17,21,28, 32,38]. A study exploring staff trauma experiences in LMICs underlined that organisational diversity across the region led to limited uniform policies which reduced communication among trauma facilities [10].

For peer review only - http://bmjopen.bmj.com/site/about/guidelines.xhtml

Domain 3: Inner Setting

Increased staff education and training strengthened the implementation climate of quality improvement interventions [18,19,20,21,24,25,29,35,36,39,41]. An integrated self-management support paper emphasised successful training as accessible to staff at all levels, with a focus on flexibility [24]. Professional hierarchies acted as a barrier to the continued education of all staff [10,19,24,25]. A training program clinician survey found that traditional decision-making hierarchies acted as a major barrier to effective education as team members were *"reluctant to speak up to highlight a problem, clarify information, or question a senior's decision when they were concerned"* [25 p.1151].

Implementation readiness was positively impacted by prioritising leadership buy-in [10,17,20,35,39]. An electronic registry in Pakistan highlighted obtaining senior management buy-in as a catalyst to improving trauma care quality [39]. Lack of stakeholder buy-in acted as a barrier to achieving progress in trauma care [17,21]. A trauma registry custodian from an LMIC hospital stated that a common opinion was that *"even basic care is not being given to the patients…how are we going to sort of spend for a registry?"* [21 p.122].

Diversifying resource sources is fundamental for successful implementation [17,29,42]. Respondents in a resource-constrained setting highlighted demand should be met by broadening the registry scope to be eligible for more grants [17]. This was in response to a common barrier around limited resources [17,18,21,23,29,36,39,40,42]. A trauma registry custodian explained that staff must *"make do with the resources that you have because the funding will not be there"* [23 p.120].

Domain 4: Characteristics of Individuals

Generating staff understanding of quality improvement is needed to encourage change [10,20,21]. Increased quality improvement dissemination can help develop greater public awareness [10,20]. A barrier to this effort is the lack of staff belief in quality improvement interventions [10,16,17,30]. A participant from a healthcare provider interview on implementing the WHOTCC highlighted

For peer review only - http://bmjopen.bmj.com/site/about/guidelines.xhtml

that *"nobody is really trying to do it as a career"* as the majority *"would rather be doing joint replacements, rather than worrying about the poor people falling off trains and motorcycles"* [30 p.19].

Communication training was a facilitator for TQIP implementation [16,24,25,31,32,34]. A trauma training clinician survey emphasised developing interpersonal staff communication alongside strengthening emergency communication methods [32]. Limited staff knowledge acts as a hurdle to improved communication [10,26,31,34]. A subject from a US injury prevention program highlighted the lack of healthy understanding of quality improvement requirements as they are viewed as *"the black sheep"* of the department, making it a constant struggle to *"seek out other mentors of people that do similar work"* [26 p.339].

Domain 5: Process of Implementation

Identifying staff to act as a champion for the quality improvement intervention is significant [17,26,30,36]. Trauma registry questionnaire respondents felt that limited stakeholder engagement could be overcome by having a champion to drive and mobilise buy-in [17]. Identifying a champion is made difficult by the barrier of overworked staff [10,16,18,31,17,34,37]. A Tanzanian hospital was so understaffed that *"you will find you have one doctor at night and ten patients show up at once"*, making it difficult to document records and motivate staff [37 p.26].

Strengthening data quality was highlighted as a facilitator [16,19,20,21,39,40,41,42]. Asia-Pacific trauma leaders proposed establishing standardised minimum data requirements to collect more complete patient information and integrate injury surveillance with data collection [19]. These efforts were hindered by poor data quality across country income levels [17,18,19,21,23,32,41,42]. An Argentinian registry implementation project found a large variation in trauma admission protocols and widespread under-reporting, impacting the quality of clinician records [32].

Common facilitators and barriers were arranged by income level and are summarised in table 3.

For peer review only - http://bmjopen.bmj.com/site/about/guidelines.xhtml

Table 3: Main facilitators and barriers to TQIP implementation by income level

Income Level	Facilitators	Barriers
L	• Staff education & training<br>• Open dialogue with key stakeholders<br>• Strengthening data quality	• Poor Data Quality<br>• Limited Resources<br>• Overworked Staff
LM	• Staff education & training<br>• Tailoring to local context<br>• Strengthening data quality	• Poor data quality<br>• Lack of buy-in<br>• Lack of common guidelines
UM	• Staff education & training<br>• Tailoring to local context<br>• Prioritising buy-in	• Poor data quality<br>• Lack of common guidelines<br>• Lack of staff knowledge
H	• Staff education & training<br>• Communication training<br>• Prioritising patient care	• Professional hierarchies<br>• Lack of common guidelines<br>• Overworked staff

Discussion:

In-hospital TQIP facilitators and barriers were similar across all income levels. Facilitators and barriers, grouped under the five CFIR domains, identified prioritising staff education and training, strengthening dialogue with stakeholders, and increasing standardised guidelines for best practice as key facilitators going forward. Major barriers were the need to prioritise data quality improvement in LMICs and improved communication training in HICs. Studies focusing on in-hospital TQIPs in LMICs were limited to a few countries, indicating that the geographic scope of quality improvement efforts must be widened in these regions.

For peer review only - http://bmjopen.bmj.com/site/about/guidelines.xhtml

A facilitator was strengthening stakeholder dialogue to increase buy-in for in-hospital TQIPs. This supports a previous qualitative interview study that proposed adopting a hybrid quality improvement model where experts would set basic guidelines for programs, incorporating feedback from frontline staff members according to local conditions [43]. Key barriers to stakeholder dialogue included a limited desire for TQIPs, resistance to change, and competing group interests. Studies reported the need to tailor interventions to the local context, along with focusing on simplicity to improve communication. An earlier grounded theory analysis of HIC interviews identified important strategies to improve stakeholder engagement as clarity of purpose, inviting participation, and engaging clinicians with feedback [44]. Given the wide range of competing innovations in hospital settings, focusing on increasing buy-in through stakeholder dialogue could strengthen the likelihood of effective implementation along with positive patient outcomes.

A reported barrier across all country income levels was the lack of common guidelines throughout the trauma care timeline. A review of LMIC trauma registry implementation highlighted that the WHO's *Injury Surveillance Guidelines* are only focused on collecting data on injury events rather than also addressing hospital care or trauma outcomes [45]. The uncoordinated and resource-intensive nature of data collection acted as a barrier that contributed to staff time constraints. A survey revealed that there is no single resource available that provides a comprehensive insight into experiences across trauma registries [46]. To overcome this, quality improvement guideline standardisation was emphasised as a facilitator to improve the success of in-hospital implementation.

Limited in-hospital TQIP durability data underlines the need to prioritise research on sustainable interventions. An LMIC scoping review on trauma registry implementation highlighted the need to prioritise sustainable registry development as a significant step forward [45]. Key barriers reported were the lack of long-term funding and limited buy-in for quality improvement initiatives. In our review, financial resources were not reported as widely as initially expected, particularly in LMICs. Despite this, key identified barriers such as staff time constraints and limited resources are directly linked to a lack of funding. Increased dissemination of results is a critical facilitator to establish prolonged TQIP support, through enhancing engagement and quality improvement understanding. A trauma care provider survey in the Andean region concluded that increased

For peer review only - http://bmjopen.bmj.com/site/about/guidelines.xhtml

quality improvement dissemination can empower local providers to participate in health systems and drive change [37]. The increase in peer-reviewed quality improvement reports from LMICs emphasises dissemination through publications as a viable means of providing guidance for future programs and expanding quality improvement knowledge [47].

Education and training are essential facilitators to improve the chances of successful implementation. This was mentioned at all country income levels, specifically in terms of interpersonal communication in HICs. Enhancing communication channels between trauma teams to improve health outcomes is increasingly common in HICs. A quantitative study found that well-defined interdisciplinary communication between the trauma service and surgical specialists reduced the time-to-operation for facial trauma patients [48]. Common barriers were limited staff belief and knowledge in quality improvement interventions. While elements of the WHO's guidelines for TQIPs have been mandated by governments across income levels [49], there have not been clear protocols put into place for implementing, evaluating, and sustaining interventions. This makes it difficult to provide standardised training for staff, which can impact the quality of patient care.

Enhancing data quality is a crucial facilitator to strengthening in-hospital implementation. Trauma registries were the most common in-hospital TQIP intervention, particularly in LMICs, with studies citing poor data quality as the main barrier. Reasons for the lack of data quality centered on high rates of missing data and limited standardised variables for comparison. A scoping review assessing trauma registry data quality found that only four out of sixty-nine publications provided a general classification of data quality, in which the taxonomy was inconsistent [50]. A similar systematic review identified only ten studies that evaluated data quality in trauma registries [51]. The majority assessed data quality based on completeness, with large differences reported between papers, and no evidence found on data precision or timeliness [51]. This lack of uniformity makes it difficult to draw insights on the quality of trauma care delivered and compare across country income levels. The Lancet Global Health Commission on High Quality Health Systems underlined the disparity between the growing global injury burden and the limited data availability on care quality [52]. Providing incentives for data entry participation, such as hospital-specific dashboards

For peer review only - http://bmjopen.bmj.com/site/about/guidelines.xhtml

or research involvement, could improve data quality and support effective in-hospital implementation.

Strengths and Limitations:

The review covered a broad range of in-hospital TQIP interventions and included studies with any design. The screening process was conducted along with two peer reviewers, which allowed for comparison and greater input in decision-making – increasing protection against potential bias This review provides a snapshot of the research field through a methodologically robust process which includes a transparent method of reviewing in a limited timeframe [53]. The review captured a large number of studies for analysis from various databases.

The use of three databases and the restriction of literature to English-language studies could have biased the findings if excluded studies were significantly different to those included in the scoping review. The use of more databases, including preprint servers, a wider timeframe, and the inclusion of a greater number of studies could have provided data that may have been neglected. We attempted to limit the selection bias by consulting a librarian with extensive experience conducting scoping reviews when selecting databases.

Grouping literature into income levels by country may provide another limitation. This classification does not capture the complexity of resource allocation within countries, reducing the generalisability of the results. The scoping review process does not place as much emphasis on assessing the quality of included studies when compared with systematic reviews [54]. The lack of quality assessment limits the implications of gathered data as the study focus is primarily concerned with mapping available literature on the topic. There is a risk of researcher bias as the process of choosing and analysing literature can be subjective. Important steps to avoid researcher bias were focusing on the research question, following the eligibility criteria, and cross-referencing screening selection with a peer–reviewer.

For peer review only - http://bmjopen.bmj.com/site/about/guidelines.xhtml

Conclusion:

Staff education and training, strengthening stakeholder dialogue, and increasing standardised trauma care guidelines were reported as the main facilitators, while major barriers identified were poor data quality in LMICs and the lack of effective communication training in HICs. Stakeholder prioritisation of TQIPs, along with increased knowledge and consensus on trauma care best practice could further advance efforts to lower the global trauma burden. The focus of future in-hospital TQIPs in LMICs should primarily be concerned with improving the data quality of registries, while interventions in HICs should focus on the communication skills of healthcare professionals.

List of abbreviations:

Abbreviation	Meaning
LMIC	Low and middle-income country
TQIP	Trauma Quality Improvement Program
CFIR	Consolidated Framework for Implementation Research
HIC	High-income country
WHO	World Health Organisation

Figure Legends:

Figure 1: CFIR Conceptual Framework Outline adapted from Damschroder et al. 2009 [13]

Figure 2: Study selection according to PRISMA ScR statement

For peer review only - http://bmjopen.bmj.com/site/about/guidelines.xhtml

References:

1. Gerdin M. The risk of dying: predicting trauma mortality in urban Indian hospitals [dissertation on the internet]. Stockholm: Karolinska Institutet; 2015 [cited 2022 Mar 23]. Available from: https://openarchive.ki.se/xmlui/bitstream/handle/10616/44832/Thesis_Martin_Gerdin.pdf
2. Vos T, Lim SS, Abbafati C, Abbas KM, Abbasi M, Abbasifard M, et al. Global burden of 369 diseases and injuries in 204 countries and territories, 1990–2019: a systematic analysis for the Global Burden of Disease Study 2019. Lancet. 2020 Oct 17;396(10258):1204-22.
3. Mock C, Juillard C, Brundage S, Goosen J, Joshipura M. Guidelines for trauma quality improvement programmes. Geneva: World Health Organization, 2009.
4. Cole E, Lecky F, West A, Smith N, Brohi K, Davenport R, et al. The impact of a pan-regional inclusive trauma system on quality of care. Ann Surg. 2016; 264(1):188–94.
5. Itri JN, Bakow E, Probyn L, Kadom N, Duong PA, Gettle LM, et al. The science of quality improvement. Acad Radiol. 2017 Mar;24(3):253-62.
6. Pope C, Van Royen P, Baker R. Qualitative methods in research on healthcare quality. Qual Saf Health Care. 2002 Jun;11(2):148-52.
7. Alexander JA, Weiner BJ, Shortell SM, Baker LC, Becker MP. The role of organizational infrastructure in implementation of hospitals' quality improvement. Hosp Top. Winter 2006;84(1):11-21.
8. Alexander JA, Hearld LR. The science of quality improvement implementation: developing capacity to make a difference. Med care. 2011 Dec; 49 Suppl:S6-20.
9. Reynolds TA, Stewart B, Drewett I, Salerno S, Sawe HR, Toroyan T, et al. The impact of trauma care systems in low-and middle-income countries. Annu Rev Public Health. 2017 Mar 20;38:507-32.
10. *LaGrone LN, Fuhs AK, Egoavil EH, Rodriguez Castro MJ, Valderrama R, IsquithDicker LN, et al. Mixed-methods assessment of trauma and acute care surgical quality improvement programs in Peru. World J Surg. 2017 Apr;41(4):963-969. *
11. Tricco AC, Lillie E, Zarin W, O'Brien KK, Colquhoun H, Levac D,et alS. PRISMA extension for scoping reviews (PRISMA-ScR): checklist and explanation. Ann Intern Med. 2018 Oct 2;169(7):467-473.

For peer review only - http://bmjopen.bmj.com/site/about/guidelines.xhtml

12. WDI - The World by Income and Region [Internet]. Datatopics.worldbank.org. 2022 [cited 2022 April 25]. Available from: https://datatopics.worldbank.org/world-development-indicators/the-world-by-income-and-region.

13. Damschroder LJ, Aron DC, Keith RE, Kirsh SR, Alexander JA, Lowery JC. Fostering implementation of health services research findings into practice: a consolidated framework for advancing implementation science. Implement Sci. 2009 Aug 7; 4:50.

14. CFIR Research Team-Center for Clinical Management Research. Consolidated Framework for Implementation Research [Internet]. Ann Arbor: CFIR Research Team-Center for Clinical Management Research; 2022. [cited 2022 April 12]. Available from: https://cfirguide.org/

15. Damschroder LJ, Lowery JC. Evaluation of a large-scale weight management program using the consolidated framework for implementation research (CFIR). Implement Sci. 2013 May 10; 8:51.

16. *Croke K, Chokotho L, Milusheva S, Bertfelt J, Karpe S, Mohammed M, et al. Implementation of a multi-center digital trauma registry: Experience in district and central hospitals in Malawi. Int J Health Plann Manage. 2020 Sep;35(5):1157-1172. *

17. *Rosenkrantz L, Schuurman N, Arenas C, Jimenez MF, Hameed MS. Understanding the barriers and facilitators to trauma registry development in resource-constrained settings: a survey of trauma registry stewards and researchers. Injury. 2021 Aug;52(8):2215-2224. *

18. *Hamadani F, Razek T, Massinga E, Gupta S, Muataco M, Muripiha P, et al. Trauma surveillance and registry development in Mozambique: results of a 1-year study and the first phase of national implementation. World J Surg. 2019 Jul;43(7):1628-1635. *

19. *Stelfox HT, Joshipura M, Chadbunchachai W, Ellawala RN, O'Reilly G, Nguyen TS, et al. Trauma quality improvement in low and middle income countries of the Asia– Pacific region: a mixed methods study. World J Surg. 2012 Aug;36(8):1978-1992. *

20. *Stevens KA, Paruk F, Bachani AM, Wesson HH, Wekesa JM, Mburu J,et al. Establishing hospital-based trauma registry systems: lessons from Kenya. Injury. 2013 Dec;44 (Suppl 4): S70-S74. *

21. *O'Reilly GM, Gabbe B, Braaf S, Cameron PA. An interview of trauma registry custodians to determine lessons learnt. Injury. 2016 Jan;47(1):116-1. *

For peer review only - http://bmjopen.bmj.com/site/about/guidelines.xhtml

22. *Wiertsema SH, Donker MH, van Dongen JM, Geleijn E, Bloemers FW, Ostelo RW, et al. The Transmural Trauma Care Model can be implemented well but some barriers 46 and facilitators should be considered during implementation: a mixed methods study. J Physiother. 2021 Oct;67(4):298-307. *
23. *Hashmi ZG, Haider AH, Zafar SN, Kisat M, Moosa A, Siddiqui F, et al. Hospitalbased trauma quality improvement initiatives: first step toward improving trauma outcomes in the developing world. J Trauma Acute Care Surg. 2013 Jul;75(1):60-68. *
24. *Hollinshead L, Jones F, Silvester L, Marshall-Taylor P. Implementing an integrated approach to self-management support in an acute major trauma therapy team: an improvement project. BMJ Open Qual. 2019 Jul 4;8(3): e000415. *
25. *Murphy M, Curtis K, McCloughen A. Facilitators and barriers to the clinical application of teamwork skills taught in multidisciplinary simulated trauma team training. Injury. 2019 May;50(5):1147-1152. *
26. *Newcomb AB, Zadnik M, Carlini AR, Francis MM, Frey KP, Heins SE, et al. Barriers and facilitators to the implementation of injury prevention programs: a qualitative exploration and model development. J Trauma Nurs. 2020;27(6):335-345. *
27. *LaGrone LN, Pozo DA, Figueroa JF, Artunduaga MA, Egoavil EH, Castro MJ, et al. Status of trauma quality improvement programs in the Andean region: what foundation do we have to build on. Injury. 2017 Sep;48(9):1985-1993. *
28. *Rosenkrantz L, Schuurman N, Arenas C, Nicol A, Hameed MS. Maximizing the potential of trauma registries in low-income and middle-income countries. Trauma Surg Acute Care Open. 2020 May 12;5(1): e000469.*
29. *Parreira JG, Campos TD, Perlingeiro JA, Soldá SC, Assef JC, Gonçalves AC, et al. Implementation of the trauma registry as a tool for quality improvement in trauma care in a Brazilian hospital: the first 12 months. Rev Col Bras Cir. 2015 Jul;42(4):265- 272. *
30. *Wild H, Mock C, Lim A. Implementation of the WHO Trauma Care Checklist: A qualitative analysis of facilitators and barriers to use. Int J Surg. 2020 Nov; 83:15-23. *
31. *Fuangworawong P, LaGrone LN, Chadbunchachai W, Sornsrivichai V, Mock CN, Vavilala MS. Assessment of trauma quality improvement activities at public hospitals in Thailand. Int J Surg. 2016 Sep;33 Pt A:88-95. *

For peer review only - http://bmjopen.bmj.com/site/about/guidelines.xhtml

32. *Monteverde E, Santero M, Bosque L, Lartigue B, Tenaillon C, Baliña J, et al. A public–private collaborative model for a trauma program implementation: findings from a prospective trauma registry at 14 hospitals in Buenos Aires, Argentina. Eur J Trauma Emerg Surg. 2021 Dec;47(6):1931-1937. *
33. *Schuurman N, Cinnamon J, Matzopoulos R, Fawcett V, Nicol A, Hameed SM. Collecting injury surveillance data in low-and middle-income countries: The Cape Town Trauma Registry pilot. Glob Public Health. 2011;6(8):874-889. *
34. *Sawe HR, Sirili N, Weber E, Coats TJ, Reynolds TA, Wallis LA. Perceptions of health providers towards the use of standardised trauma form in managing trauma patients: a qualitative study from Tanzania. Inj Epidemiol. 2020 May 1;7(1):15. *
35. *Wainiqolo I, Kafoa B, McCaig E, Kool B, McIntyre R, Ameratunga S. Development and piloting of the Fiji injury surveillance in hospitals system (TRIP Project-1). Injury. 2013 Jan;44(1):126-131. *
36. *Zargaran E, Schuurman N, Nicol AJ, Matzopoulos R, Cinnamon J, Taulu T, et al. The electronic Trauma Health Record: design and usability of a novel tablet-based tool for trauma care and injury surveillance in low resource settings. J Am Coll Surg. 2014 Jan;218(1):41-50. *
37. *Sawe HR, Sirili N, Weber E, Coats TJ, Wallis LA, Reynolds TA. Barriers and facilitators to implementing trauma registries in low-and middle-income countries: qualitative experiences from Tanzania. Afr J Emerg Med. 2020;10(Suppl 1): S23-S28.*
38. *Zhou J, Wang T, Belenkiy I, Hardcastle TC, Rouby JJ, Jiang B. Management of severe trauma worldwide: implementation of trauma systems in emerging countries: China, Russia and South Africa. Crit Care. 2021 Aug 9;25(1):286. *
39. *Mehmood A, Razzak JA, Kabir S, MacKenzie EJ, Hyder AA. Development and pilot implementation of a locally developed Trauma Registry: lessons learnt in a low-income country. BMC Emerg Med. 2013 Dec 21;13:4. *
40. *St-Louis E, Paradis T, Landry T, Poenaru D. Factors contributing to successful trauma registry implementation in low-and middle-income countries: a systematic review. Injury. 2018 Dec;49(12):2100-2110. *
41. *Chokotho LC, Mulwafu W, Nyirenda M, Mbomuwa FJ, Pandit HG, Le G, et al. Establishment of trauma registry at Queen Elizabeth Central Hospital (QECH), Blantyre,

For peer review only - http://bmjopen.bmj.com/site/about/guidelines.xhtml

Malawi and mapping of high-risk geographic areas for trauma. World J Emerg Med. 2019;10(1):33-41. *

42. *Bommakanti K, Feldhaus I, Motwani G, Dicker RA, Juillard C. Trauma registry implementation in low-and middle-income countries: challenges and opportunities. J Surg Res. 2018 Mar; 223:72-86. *
43. Parker LE, Kirchner JE, Bonner LM, Fickel JJ, Ritchie MJ, Simons CE, et al. Creating a quality-improvement dialogue: utilising knowledge from frontline staff, managers, and experts to foster health care quality improvement. Qual Health Res. 2009 Feb;19(2):229-242
44. Santana MJ, Straus S, Gruen R, Stelfox HT. A qualitative study to identify opportunities for improving trauma quality improvement. J Crit Care. 2012 Dec;27(6):738.e1-7.
45. Rosenkrantz L, Schuurman N, Hameed M. Trauma registry implementation and operation in low and middle income countries: a scoping review. Glob Public Health. 2019 Dec;14(12):1884-1897.
46. O'Reilly GM, Gabbe B, Cameron PA. Trauma registry methodology: a survey of trauma registry custodians to determine current approaches. Injury. 2015 Feb;46(2):201-206.
47. Sifrim ZK, Barker PM, Mate KS. What gets published: the characteristics of quality improvement research articles from low-and middle-income countries. BMJ Qual Saf. 2012 May;21(5):423-431
48. Keller PR, Schneeberger S, Drolet BC, Al Kassis S, Pearce FB, Perdikis G. Implementation of a trauma quality improvement program communications package decreases time-to-operation for facial trauma. Ann Plast Surg. 2019 Jun;82(6S Suppl 5):S380-S385.
49. Bardfield J, Agins B, Akiyama M, Basenero A, Luphala P, Kaindjee-Tjituka F, et al. A quality improvement approach to capacity building in low-and middle-income countries. Aids. 2015 Jul;29(Suppl 2):S179-S186.
50. O'Reilly GM, Gabbe B, Moore L, Cameron PA. Classifying, measuring and improving the quality of data in trauma registries: a review of the literature. Injury. 2016 Mar;47(3):559-567.
51. Porgo TV, Moore L, Tardif PA. Evidence of data quality in trauma registries: a systematic review. J Trauma Acute Care Surg. 2016 Apr;80(4):648-658.

For peer review only

52. Whitaker J, Nepogodiev D, Leather A, Davies J. Assessing barriers to quality trauma care in low and middle-income countries: a Delphi study. Injury. 2020 Feb;51(2):278- 285
53. Gosselin RA, Spiegel DA, Coughlin R, Zirkle LG. Injuries: the neglected burden in developing countries. Bull World Health Organ. 2009 Apr;87(4):246-246a.
54. Arksey H, O'Malley L. Scoping studies: towards a methodological framework. Int J Soc Res Methodol. 2005 Feb;8(1):19-32.

Note: * studies included in the review*

Acknowledgements:

We would like to acknowledge Karolinska Institutet Library (Independent Information Specialist Consultants, Solna, Sweden) for peer review of the literature search strategy, along with the faculty and students of Karolinska Institutet Department of Global Public Health for comments and feedback on earlier versions of this manuscript.

Funding

No funding sources used

Availability of data and materials

The data that support the findings of this study are available from PubMed, Web of Science, and Global Index Medicus databases using the search string outlined in Online Supplement 1.

Authors' contributions

Study concept, design and search strategy: GK, JB, MGW. Data selection and screening: GK, JB, YS, MGW. Data analysis and interpretation: GK, JB, MGW. Drafting of manuscript: GK, JB, MGW. Critical revisions of manuscript: GK, JB, YS, MGW. Study supervision: JB, MGW. All authors read and approved the final manuscript.

Ethics Declarations:

For peer review only - http://bmjopen.bmj.com/site/about/guidelines.xhtml

Ethics approval and consent to participate

Not applicable

Consent for publication

Not applicable

Competing interests

The authors declare that they have no competing interests

For peer review only

For peer review only - http://bmjopen.bmj.com/site/about/guidelines.xhtml

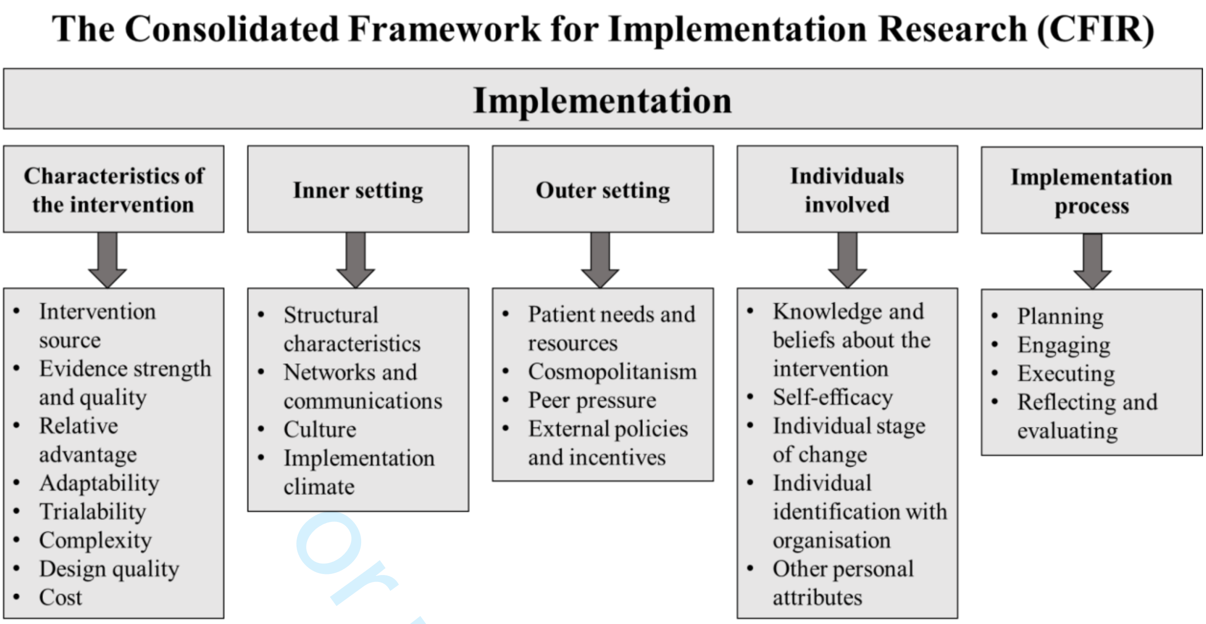

For peer review only

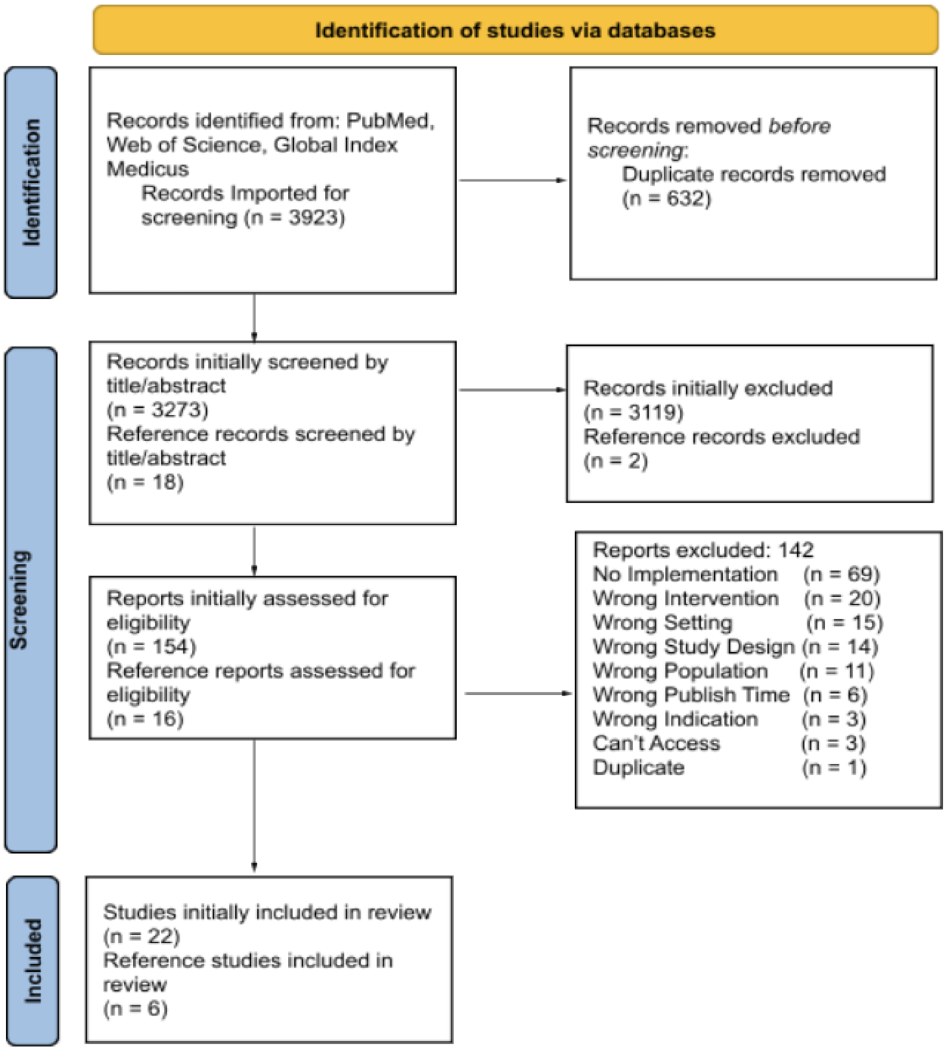
Identification of studies via databases
Identification
Records identified from: PubMed, Web of Science, Global Index Medicus
Records Imported for screening (n = 3923)
Records removed *before screening*:
Duplicate records removed (n = 632)
Screening
Records initially screened by title/abstract (n = 3273)
Reference records screened by title/abstract (n = 18)
Records initially excluded (n = 3119)
Reference records excluded (n = 2)
Reports initially assessed for eligibility (n = 154)
Reference reports assessed for eligibility (n = 16)
Reports excluded: 142
No Implementation (n = 69)
Wrong Intervention (n = 20)
Wrong Setting (n = 15)
Wrong Study Design (n = 14)
Wrong Population (n = 11)
Wrong Publish Time (n = 6)
Wrong Indication (n = 3)
Can't Access (n = 3)
Duplicate (n = 1)
Included
Studies initially included in review (n = 22)
Reference studies included in review (n = 6)

Online Supplement:

Online Supplement 1: Database search strategy according to Population, Concept, Context (PCC) framework

**Population:**		
**#1**	**MeSH terms:**	Humans and Trauma Centres
**#2**	**Free Text:**	Population OR populations OR patient OR patients OR adult OR adults OR injured OR victim OR victims OR trauma centre OR trauma centres OR trauma department OR trauma departments OR trauma facility OR trauma facilities OR acute care centre OR acute care centres OR acute care department OR acute care departments OR acute care facility OR acute care facilities OR primary care centre OR primary care centres OR primary care department OR primary care departments OR primary care facility OR primary facilities OR emergency centre OR emergency centres OR emergency department OR emergency departments OR emergency facility OR emergency facilities
**#3**	**#1 OR #2**	
**Concept:**		
**#4**	**MeSH terms:**	Hospital and quality improvement
**#5**	**Free Text:**	Hospital OR hospitals OR in-hospital OR hospital-based OR facility OR facilities AND quality improvement OR quality indicator OR facilitator OR facilitators OR opportunity OR opportunities OR advantage OR advantages OR solution OR solutions OR barrier OR barriers OR challenge OR challenges OR disadvantage OR disadvantages OR inhibitor OR inhibitors OR implementation OR intervention OR innovation
**#6**	**#4 OR #5**	
**Context:**		
**#7**	**MeSH terms:**	Developing countries and developed countries
**#8**	**Free Text:**	Developing country OR developing countries OR developing nation OR developing nations OR developing economy OR developing economies OR low income country OR low income countries OR low income nation OR low income nations OR low income economy OR low income economies OR lower middle income

		country OR lower middle income countries OR lower middle income nation OR lower middle income nations OR lower middle income economy OR lower middle income economies OR upper middle income country OR upper middle income countries OR upper middle income nation OR upper middle income nations OR upper middle income economy OR upper middle income economies OR middle income country OR middle income countries OR middle income nation OR middle income nations OR middle income economy OR middle income economies OR transitional country OR transitional countries OR transitional nation OR transitional nations OR transitional economy OR transitional economies OR developed country OR developed countries OR developed nation OR developed nations OR developed economy OR developed economies OR high income country OR high income countries OR high income nation OR high income nations OR high income economy OR high income economies
**#9**	**#7 OR #8**	
**#10**	**#3 AND #6 OR #9**	3923 Results
**Filter**	English language studies published from June 2009 onwards	

Online Supplement 2: Characteristics of extracted studies

Income Level	Continents/Countries	Source	Study Type	Intervention Type	Intervention Duration (months)
L	Malawi	Chokotho et al. 2019 [16]	Qualitative - Survey	T.R	N/A
L	Malawi	Croke et al. 2020 [17]	Qualitative - Survey	T.R	N/A
L	Mozambique	Hamadani et al. 2019 [18]	Qualitative - Interview	T.R	12
L	Tanzania[a]	Sawe et al. 2020 [19]	Qualitative - Focus Group Discussion	T.R	N/A
L	Tanzania[a]	Sawe et al. 2020 [20]	Qualitative - Interview	T.R	N/A
LM	Pakistan	Hashmi et al. 2013 [21]	Quantitative - logistic regression	T.R, Q.A.P	108
LM	Pakistan	Mehmood et al. 2013 [22]	Qualitative - Survey	T.R	3

For peer review only

LM	Asia: 7	Stelfox et al. 2012 [23]	Mixed Methods	Range - M&M, PDP, AF, QOC Audit, Statistical Analysis	N/A
LM	Kenya	Stevens et al. [24]	Qualitative - Survey	T.R	N/A
UM	Thailand	Fuangworawong et al. 2016 [25]	Qualitative - Survey	Range - M&M, T.R, A.F, PDP	N/A
UM	Peru	LaGrone et al. 2017 [10]	Mixed methods	Range - M&M/T.R	N/A
UM	Argentina	Monteverde et al. 2021 [26]	Qualitative - Univariate/Bivariate descriptive analysis	T.R	114
UM	Brazil	Parreira et al. 2015 [27]	Qualitative - Survey	T.R	12
UM	South Africa	Schuurman et al. 2011 [28]	Qualitative - Survey	T.R	1
UM	Fiji	Wainiqolo et al. 2013 [29]	Qualitative - Survey	T.R	5
UM	Africa: 1, Asia: 1, Europe: 1	Zhou et al. 2021 [30]	Literature Review	T.R	N/A
H	U.K	Hollinshead et al. 2019 [31]	Qualitative - Survey	Educational - B.S.M.P	12
H	Australia	Murphy et al. 2019 [32]	Qualitative - Survey	Multidisciplinary TTT	N/A
H	U.S.A	Newcomb et al. 2020 [33]	Qualitative - Interview	I.P.P	N/A
H	Netherlands	Wiertsema et al. 2021 [34]	Mixed Methods	TTCM	N/A
Range - LMIC	Africa: 6, Asia: 3, South America: 3, North America: 1	Rosenkrantz et al. 2021 [35]	Qualitative - Survey	T.R	Range - 3 = <24 1 = 24-60, 7 = 72-120, 2 = >120
Range - LMIC	Africa: 9, Asia: 4, South	St-Louis et al. 2018 [36]	Literature Review	T.R	N/A

For peer review only

	America: 3, North America: 1, Oceania: 1				
Range - LM/UM	South America: 4	LaGrone et al. 2017 [37]	Qualitative - Survey	Range - M&M, T.R, A.F, Autopsies	N/A
Range - UM/H	South Africa/Canada	Zargaran et al. 2014 [38]	Qualitative - Survey	T.R	1
Range - All	Africa: 6, Asia: 6, North America: 3, South America: 2, Europe: 1, Oceania: 1	Bommakanti et al. 2018 [39]	Literature Review	T.R	N/A
Range - All	Africa: 8, Asia: 6, Europe: 3, North America: 3, Oceania: 2	O'Reilly et al. 2016 [40]	Qualitative - Interview	T.R	N/A
Range - All	Asia: 5, Africa: 3, South America: 1, North America: 1, Oceania: 1	Wild et al. 2020 [41]	Qualitative - Interview	TCC	24
Range - Unspecified	Africa: 7, Asia: 3, North America: 2, Europe: 1, Oceania: 1	Rosenkrantz et al. 2020 [42]	Literature Review	T.R	N/A

Note. L = Low Income, LM = Lower Middle Income, UM = Upper Middle Income, H = High Income, T.R = Trauma Registry, Q.A.P = Quality Assurance Program, M&M = Mortality & Morbidity Conferences, PDP = Preventable Death Panel, A.F = Audit Filter, QOC = Quality of Care, B.S.M.P = Bridges Self-Management Programme, TTT = Trauma Team Training, I.P.P = Injury Prevention Program, TTCM = Transmural Trauma Care Model, TCC = Trauma Care Checklist.

"Qualitative - Survey" design included studies that conducted questionnaires

[a]Tanzania reclassified from low to lower middle income country after data collection of studies

For peer review only

Preferred Reporting Items for Systematic reviews and Meta-Analyses extension for Scoping Reviews (PRISMA-ScR) Checklist

SECTION	ITEM	PRISMA-ScR CHECKLIST ITEM	REPORTED ON PAGE #
**TITLE**			
Title	1	Identify the report as a scoping review.	1
**ABSTRACT**			
Structured summary	2	Provide a structured summary that includes (as applicable): background, objectives, eligibility criteria, sources of evidence, charting methods, results, and conclusions that relate to the review questions and objectives.	2
**INTRODUCTION**			
Rationale	3	Describe the rationale for the review in the context of what is already known. Explain why the review questions/objectives lend themselves to a scoping review approach.	4
Objectives	4	Provide an explicit statement of the questions and objectives being addressed with reference to their key elements (e.g., population or participants, concepts, and context) or other relevant key elements used to conceptualize the review questions and/or objectives.	4
**METHODS**			
Protocol and registration	5	Indicate whether a review protocol exists; state if and where it can be accessed (e.g., a Web address); and if available, provide registration information, including the registration number.	5
Eligibility criteria	6	Specify characteristics of the sources of evidence used as eligibility criteria (e.g., years considered, language, and publication status), and provide a rationale.	5
Information sources*	7	Describe all information sources in the search (e.g., databases with dates of coverage and contact with authors to identify additional sources), as well as the date the most recent search was executed.	5
Search	8	Present the full electronic search strategy for at least 1 database, including any limits used, such that it could be repeated.	Online Supplement 1
Selection of sources of evidence†	9	State the process for selecting sources of evidence (i.e., screening and eligibility) included in the scoping review.	5
Data charting process‡	10	Describe the methods of charting data from the included sources of evidence (e.g., calibrated forms or forms that have been tested by the team before their use, and whether data charting was done independently or in duplicate) and any processes for obtaining and confirming data from investigators.	5
Data items	11	List and define all variables for which data were sought and any assumptions and simplifications made.	5
Critical appraisal of individual sources of evidence§	12	If done, provide a rationale for conducting a critical appraisal of included sources of evidence; describe the methods used and how this information was used in any data synthesis (if appropriate).	N/A

St. Michael's
Inspired Care.
Inspiring Science.

For peer review only - http://bmjopen.bmj.com/site/about/guidelines.xhtml

SECTION	ITEM	PRISMA-ScR CHECKLIST ITEM	REPORTED ON PAGE #
Synthesis of results	13	Describe the methods of handling and summarizing the data that were charted.	6
**RESULTS**			
Selection of sources of evidence	14	Give numbers of sources of evidence screened, assessed for eligibility, and included in the review, with reasons for exclusions at each stage, ideally using a flow diagram.	7
Characteristics of sources of evidence	15	For each source of evidence, present characteristics for which data were charted and provide the citations.	Online Supplement 2
Critical appraisal within sources of evidence	16	If done, present data on critical appraisal of included sources of evidence (see item 12).	N/A
Results of individual sources of evidence	17	For each included source of evidence, present the relevant data that were charted that relate to the review questions and objectives.	7-15
Synthesis of results	18	Summarize and/or present the charting results as they relate to the review questions and objectives.	15
**DISCUSSION**			
Summary of evidence	19	Summarize the main results (including an overview of concepts, themes, and types of evidence available), link to the review questions and objectives, and consider the relevance to key groups.	16-18
Limitations	20	Discuss the limitations of the scoping review process.	18
Conclusions	21	Provide a general interpretation of the results with respect to the review questions and objectives, as well as potential implications and/or next steps.	18-19
**FUNDING**			
Funding	22	Describe sources of funding for the included sources of evidence, as well as sources of funding for the scoping review. Describe the role of the funders of the scoping review.	25

JBI = Joanna Briggs Institute; PRISMA-ScR = Preferred Reporting Items for Systematic reviews and Meta-Analyses extension for Scoping Reviews.
* Where *sources of evidence* (see second footnote) are compiled from, such as bibliographic databases, social media platforms, and Web sites.
† A more inclusive/heterogeneous term used to account for the different types of evidence or data sources (e.g., quantitative and/or qualitative research, expert opinion, and policy documents) that may be eligible in a scoping review as opposed to only studies. This is not to be confused with *information sources* (see first footnote).
‡ The frameworks by Arksey and O'Malley (6) and Levac and colleagues (7) and the JBI guidance (4, 5) refer to the process of data extraction in a scoping review as data charting.
§ The process of systematically examining research evidence to assess its validity, results, and relevance before using it to inform a decision. This term is used for items 12 and 19 instead of "risk of bias" (which is more applicable to systematic reviews of interventions) to include and acknowledge the various sources of evidence that may be used in a scoping review (e.g., quantitative and/or qualitative research, expert opinion, and policy document).

From: Tricco AC, Lillie E, Zarin W, O'Brien KK, Colquhoun H, Levac D, et al. PRISMA Extension for Scoping Reviews (PRISMAScR): Checklist and Explanation. Ann Intern Med. 2018;169:467–473. doi: 10.7326/M18-0850.

For peer review only - http://bmjopen.bmj.com/site/about/guidelines.xhtml

Facilitators and barriers impacting in-hospital Trauma Quality Improvement Program (TQIP) implementation across country income levels: a scoping review

Journal:	*BMJ Open*
Manuscript ID	bmjopen-2022-068219.R2
Article Type:	Original research
Date Submitted by the Author:	06-Feb-2023
Complete List of Authors:	Kapanadze, George; Karolinska Institute, Global Public Health<br>Berg, Johanna; Karolinska Institute, Global Public Health; Skånes universitetssjukhus Malmö, Emergency and Internal medicine<br>Sun, Yue; Karolinska Institute, Global Public Health<br>Gerdin Wärnberg, Martin ; Karolinska Institutet, Department of Global Public Health; Karolinska University Hospital, Function Perioperative Medicine and Intensive Care
**Primary Subject Heading**:	Global health
Secondary Subject Heading:	Public health, Qualitative research
Keywords:	Quality in health care < HEALTH SERVICES ADMINISTRATION & MANAGEMENT, TRAUMA MANAGEMENT, Change management < HEALTH SERVICES ADMINISTRATION & MANAGEMENT

SCHOLARONE™ Manuscripts

I, the Submitting Author has the right to grant and does grant on behalf of all authors of the Work (as defined in the below author licence), an exclusive licence and/or a non-exclusive licence for contributions from authors who are: i) UK Crown employees; ii) where BMJ has agreed a CC-BY licence shall apply, and/or iii) in accordance with the terms applicable for US Federal Government officers or employees acting as part of their official duties; on a worldwide, perpetual, irrevocable, royalty-free basis to BMJ Publishing Group Ltd ("BMJ") its licensees and where the relevant Journal is co-owned by BMJ to the co-owners of the Journal, to publish the Work in this journal and any other BMJ products and to exploit all rights, as set out in our licence.

The Submitting Author accepts and understands that any supply made under these terms is made by BMJ to the Submitting Author unless you are acting as an employee on behalf of your employer or a postgraduate student of an affiliated institution which is paying any applicable article publishing charge ("APC") for Open Access articles. Where the Submitting Author wishes to make the Work available on an Open Access basis (and intends to pay the relevant APC), the terms of reuse of such Open Access shall be governed by a Creative Commons licence – details of these licences and which Creative Commons licence will apply to this Work are set out in our licence referred to above.

Other than as permitted in any relevant BMJ Author's Self Archiving Policies, I confirm this Work has not been accepted for publication elsewhere, is not being considered for publication elsewhere and does not duplicate material already published. I confirm all authors consent to publication of this Work and authorise the granting of this licence.

For peer review only

Facilitators and barriers impacting in-hospital Trauma Quality Improvement Program (TQIP) implementation across country income levels: a scoping review

George Kapanadze, MMSc; Johanna Berg, MD; Yue Sun, MPH; Martin Gerdin Wärnberg MD, PhD

Author Affiliations: Department of Global Public Health, Karolinska Institutet, Solna, Sweden (Kapanadze); Department of Global Public Health, Karolinska Institutet, Solna, Sweden, Department of Internal and Emergency Medicine, Skåne University Hospital , Malmö (Berg); Department of Global Public Health, Karolinska Institutet, Solna, Sweden (Sun); Department of Global Public Health, , Karolinska Institutet, Solna, Sweden; Perioperative Medicine and Intensive Care, Karolinska University Hospital, Solna, Sweden (Gerdin Wärnberg)

Correspondence to:
George Kapanadze, MMSc, Department of Global Public Health, Karolinska Institutet,Widerströmska huset Tomtebodavägen 18 A, plan 3, 171 65 Solna, Sweden
george.kapanadze@stud.ki.se

For peer review only

Abstract

Objective: Trauma is a leading cause of mortality and morbidity globally, disproportionately affecting low-income and middle-income countries (LMICs). Understanding the factors determining implementation success for in-hospital Trauma Quality Improvement Programs (TQIPs) is critical to reducing the global trauma burden. We synthesised topical literature to identify key facilitators and barriers to in-hospital TQIP implementation across country income levels.

Design: Scoping review.

Data sources: PubMed, Web of Science, and Global Index Medicus databases were searched from June 2009 – January 2022.

Eligibility criteria: Published literature involving any study design, written in English, and evaluating any implemented in-hospital quality improvement program in trauma populations worldwide. Literature that was non-English, unpublished and involved non-hospital TQIPs was excluded.

Data extraction and synthesis: Two reviewers completed a three-stage screening process using Covidence, with any discrepancies resolved through a third reviewer. Content analysis using the Consolidated Framework for Implementation Research (CFIR) identified facilitator and barrier themes for in-hospital TQIP implementation.

Results: Twenty-eight studies met the eligibility criteria from 3923 studies identified. The most discussed in-hospital TQIP in included literature were trauma registries. Facilitators and barriers were similar across all country income levels. The main facilitator themes identified were the prioritisation of staff education and training, strengthening stakeholder dialogue, and providing standardised best-practice guidelines. The key barrier theme identified in LMICs was poor data quality, while high-income countries (HICs) had reduced communication across professional hierarchies.

Conclusions: Stakeholder prioritisation of in-hospital TQIPs, along with increased knowledge and consensus of trauma care best practices are essential efforts to reduce the global trauma burden. The primary focus of future studies on in-hospital TQIPs in LMICs should target improving registry data quality, while interventions in HICs should target strengthening communication channels between healthcare professionals.

For peer review only

For peer review only - http://bmjopen.bmj.com/site/about/guidelines.xhtml

Strengths and limitations of this study

- The present scoping review was based on a comprehensive search of published literature that included a robust screening process involving multiple peer reviewers to protect against bias.
- We used the Consolidated Framework for Implementation Research (CFIR) to ground collected evidence in an established theory.
- The generalisability of our results was impacted by grouping literature based on country income level, as we did not investigate potential differences in available resources within countries (e.g., regions and healthcare institutions).
- The lack of quality assessment involved in scoping review design limits the implications of gathered results, given the focus on mapping available literature, and identifying knowledge gaps.

Introduction

Trauma is the clinical entity composed of physical injury and the body's associated response [1]. Trauma is a leading cause of death and disability worldwide [2]. To improve in-hospital care, multiple Trauma Quality Improvement Programs (TQIPs) have been developed, typically originating in high-income countries (HICs). Key in-hospital TQIPs highlighted by the World Health Organisation (WHO) in their 2009 guidelines included techniques such as trauma registries along with mortality and morbidity conferences (3). The implementation of TQIPs have been attributed to significant improvements in care quality and outcomes in HICs [4].

Quality Improvement is designed to achieve positive change in a specific process [5], which can be highly dependent on the perspectives, attitudes, and behaviours of patients and healthcare professionals in their local context [6]. Although quality improvement aims to improve the quality of care, organisations often struggle with its implementation [7]. While implementation research has improved significantly, there remain substantial gaps in knowledge and understanding of

For peer review only - http://bmjopen.bmj.com/site/about/guidelines.xhtml

factors that facilitate or impede efforts to implement quality improvement in healthcare organisations [8].

Despite research on developing in-hospital TQIPs steadily rising, little context-relevant guidance exists to help policymakers set priorities for implementation [9]. A recent study found a lack of in-depth examination of barriers and facilitators to TQIP implementation in low-income and middle-income countries (LMIC) [10]. This exemplifies the need for further research to assess trauma systems in LMICs to inform health system strengthening for trauma care [4]. Thus, this scoping review aimed to identify key facilitators and barriers to in-hospital TQIP implementation across country income levels.

Methods

Study design

We conducted a scoping review to map the available evidence on factors influencing in-hospital TQIP implementation. The scoping review is reported in accordance with the Preferred Reporting Items for Systematic Reviews and Meta-Analyses extension for Scoping Reviews (PRISMA-ScR) [11]. A scoping review was most appropriate due to the broad nature of the topic along with the wide variety of potential factors impacting quality improvement implementation in differing income settings. We defined country income level as the income level of each included country based on World Bank classification at the time of publication [12].

Search strategy

The search strategy was developed with a librarian using keywords, synonyms, and medical subject headings (MeSH) as shown in online supplement 1**.** The review searched three databases, *PubMed*, *Web of Science* and *Global Index Medicus*, for studies from June 2009 to January 2022. The search was limited to publications from June 2009 onwards in line with the WHO release on Guidelines for TQIPs (3).

Study selection

For peer review only - http://bmjopen.bmj.com/site/about/guidelines.xhtml

Given the review's broad scope, all in-hospital TQIPs were considered, along with studies of any design. The screening process was completed by two authors, assisted by a third reviewer. The following inclusion criteria were used:

Any English-language studies evaluating facilitators and barriers to implementation of system-wide in-hospital quality improvement programs in trauma populations worldwide.

Studies were excluded if they were:

Non-English, unpublished, if they described projects where implementation was not a focus, or if they discussed non-hospital TQIPs.

Data charting

Data extraction was performed using a predetermined form addressing study identifying information; geographic location; population and study setting; country income level at the time of publication; data collection methods; quality improvement intervention type and duration; implementation facilitators and barriers; implementation outcome and study funding sources. Appropriate quotations were identified and extracted through deductive content analysis. We did not evaluate the evidence underlying the identified TQIPs, as our focus was on facilitators and barriers to implementing a TQIP once attempted.

Summarising and reporting results

Facilitator and barrier selection was guided by the Consolidated Framework for Implementation Research (CFIR) - illustrated in figure 1. The framework is composed of five domains: intervention characteristics, outer setting, inner setting, individual characteristics, and implementation process [13]. These domains provide a practical guide for systematically assessing potential facilitators and barriers to TQIP implementation [14], while offering insights to explain differences across income levels [15].

Patient and public involvement

None.

For peer review only - http://bmjopen.bmj.com/site/about/guidelines.xhtml

Results

The literature search yielded 3923 studies, 3291 studies passed title and abstract screening while 170 reports were identified for full-text screening. The number of extracted studies was 28 [10,16-42], with details on study selection found in figure 2. Most of the studies originated in upper-middle-income countries (7 [25%]), followed by low-income (5 [18%]), high-income (4 [14%]), and lower-middle-income (4 [14%]) countries. The remaining studies (8 [29%]) included multi-country analyses that spanned across income levels. The most represented continents in the review were Africa and Asia, with South Africa (11 [39%]) and Pakistan (9 [32%]) appearing most frequently. Most studies solely discussed trauma registries as the prominent in-hospital TQIP (18 [64%]) while a small number evaluated two or more TQIPs in their reports (5 [18%]). Full study characteristic details are found in online supplement 2.

The CFIR domains identified most in the literature were intervention characteristics and inner setting. A summary of facilitators and barriers according to the CFIR are in Tables 1 and 2.

Table 1. Summary of in-hospital TQIP implementation facilitators using the CFIR

CFIR Domain	CFIR Construct	Facilitators	Quotes
Intervention Characteristics	Relative Advantage	Open dialogue with key stakeholders	1. *Active cooperation and extensive input from Ministry of Health stakeholders* [16]<br>2. *Appealing to stakeholders higher up in the Department of Health* [17]
	Adaptability	Tailoring intervention to local context	1. *All data collection was carried out by local Mozambican health workers* [18]<br>2. *Participants highlighted that patient care should be adapted to local circumstances* [19]
	Complexity	Focus on simplicity	1. *Balance between sufficient detail and simplicity of the data collection process* [20]<br>2. *Less is more when it comes to trauma registry* [21]

For peer review only - http://bmjopen.bmj.com/site/about/guidelines.xhtml

Outer Setting	Patient needs & Resources	Prioritising patient care	1. *More focus on the everyday things that patients have to deal with* [22]<br>2. *Harnessing…clinical enthusiasm to improve trauma care* [21]
	External policies & incentives	Collaboration with national/international bodies	1. *Increased focus on trauma academics through regional and international collaborations* [23]<br>2. *Seek potential collaborators with interest* [17]
Inner Setting	Implementation Climate		
	Learning Climate	Staff education & training	1. *Engage nursing and medical staff in multidisciplinary training* [24]<br>2. *Training needs to include strategies that address fundamental interpersonal communication* [25]
	Readiness for implementation		
	Leadership engagement	Prioritise buy-in	1. *Leadership was identified as having a clear impact on team factors* [25]<br>2. *The injury prevention coordinators' work was influenced by the support from direct managers and senior leadership* [26]
	Available Resources	Diversify resource sources	1. *External training resources, such as national or state conferences, were important to allow injury prevention champions to connect* [26]<br>2. *Broadening the scope of the registry to apply for additional grants* [17]
Characteristics of Individuals	Knowledge & Beliefs about the intervention	Generate understanding of quality improvement	1. *There is a need for increased dissemination of QI programs as a means of empowering local providers to participate in their health systems* [27]<br>2. *Formulate very clearly a vision, mission and strategy that everyone understands* [21]

For peer review only - http://bmjopen.bmj.com/site/about/guidelines.xhtml

	Other Personal Attributes	Communication training	1. *Important to find ways to sustain changes in clinician communication style* [24]<br>2. *Training needs to include strategies that address fundamental interpersonal communication alongside other solutions* [25]
Process of Implementation	Reflecting & Evaluating	Strengthening data quality	1. *Increasing real and perceived efficacy of mortality & morbidity conferences…through the establishment of standardised case selection criteria* [27]<br>2. *Regular audits and staged validation of data* [21]
	Engaging		
	Champions	Identifying dedicated staff	1. *Sometimes I just get inspired by something that occurs to me as nothing is being done about it currently* [26]<br>2. *Identify a person with passion and zeal to drive the effort* [17]

Table 2. Summary of in-hospital TQIP implementation barriers using the CFIR

CFIR Domain	CFIR Construct	Barriers	Quotes
Intervention Characteristics	Relative Advantage	Limited desire for quality improvement initiatives	1. *A "so what" attitude amongst stakeholders from other nearby facilities* [17]<br>2. *Current lack of motivation for quality improvement programs in Latin America* [27]
	Adaptability	Resistance to change	1. *Surgeon's preference for autonomy and self-reliance over standardisation: "I do it my way, you do it yours"* [10]<br>2. *Allied health professionals identified the barriers of…'the way things are always done"* [24]

For peer review only - http://bmjopen.bmj.com/site/about/guidelines.xhtml

	Complexity	Competing interests between groups	1. *Institutional and national prioritisation of other patient care objectives* [10]<br>2. *Trauma is a very low priority for most clinicians* [21]
Outer Setting	Patient needs & Resources	Staff time constraints	1. *Staff have multiple demands on their time* [16]<br>2. *Surgeon spends less time with a patient, he can proceed with the next patient* [22]
	External policies & incentives	Lack of common guidelines	1. *Absence of uniform policies, guidelines, and protocols* [19]<br>2. *No standards or best practices in place for the collection of data* [28]
Inner Setting	Implementation Climate		
	Learning Climate	Professional hierarchies	1. *Senior staff attempting to maintain the status quo in order to fly under the radar* [10]<br>2. *Limited ability to engage leaders* [19]
	Readiness for implementation		
	Leadership engagement	Lack of buy-in	1. *Some stakeholders…created barriers by prioritising other clinical issues* [17]<br>2. *Rest of the stakeholders and the doctors did not buy-in to this concept of using a registry* [21]
	Available Resources	Limited Resources	1. *Hospitals cannot afford to hire specialised personnel solely to collect data* [29]<br>2. *Make do with the resources that you have, because the funding will not be there* [21]
Characteristics of Individuals	Knowledge & Beliefs about the intervention	Lack of staff belief in quality improvement interventions	1. *Some initial skepticism from counterparts* [16]<br>2. *It's not fitting into anybody's career plans at this point in time…trauma happens to poor people* [30]

For peer review only - http://bmjopen.bmj.com/site/about/guidelines.xhtml

	Other Personal Attributes	Lack of staff knowledge	1. *Almost half of newly-graduated physicians do not have experience working with this QI tool during their training* [31]<br>2. *Not a healthy understanding of how we do our work or what's needed to do our work* [26]
Process of Implementation	Reflecting & Evaluating	Poor Data Quality	1. *Challenges reported included the high rates of missing data* [18]<br>2. *A major hurdle is data quality of clinical records* [32]
	Engaging		
	Champions	Overworked Staff	1. *Human resources to meet volume of data collection were underestimated* [19]<br>2. *Constant suggesting by the faculty to use overworked residents to collect trauma data* [17]

Domain 1: Characteristics of the intervention

Open dialogue with key stakeholders facilitates the implementation process [10,16,17,33]. A multi-centre digital trauma registry implementation in Malawi revealed that consistent communication between Ministry of Health stakeholders and the research team contributed to the success of the registry [16]. Competing interests between groups acted as a barrier to establishing communication channels between stakeholders [10,17,21]. The absence of a vision for trauma care was highlighted by a custodian from a multi-hospital in a developing country as there was a need to *"show the vision of better trauma care to lots of people, because trauma is a very low priority for most clinicians, and it happens to poor people"* [21 p.119].

Another key theme was the importance of adaptability. A focus on tailoring the intervention to the local context to ensure success and sustainability was apparent [10,17,18,19,25,27,34,35]. The implementation of a multi-hospital injury surveillance program in Mozambique was driven by

For peer review only - http://bmjopen.bmj.com/site/about/guidelines.xhtml

local data collectors that enhanced the sustainability of the project [18]. Tailoring programs to local conditions is often made difficult by individuals' resistance to change [10,31]. An educational self-management intervention in a trauma centre in the UK revealed that the roll-out of support was hindered by team members preferring to continue with established practices, which limited widespread adoption [24].

Attention to simplicity is an implementation facilitator [17,36,20,21]. A trauma registry development study in LMICs emphasised the significance of keeping design simple through the standardisation of variables to enhance accuracy and understanding [17]. Standardisation requires widespread staff and stakeholder support, which is often lacking due to a limited desire for quality improvement. A survey of trauma care providers in the Andean region revealed that less than half of respondents *"ever witnessed a change occur in their institution",* which influenced the *"current lack of motivation for quality improvement programs"* [27 p.1992].

Domain 2: Outer setting

Prioritising patient care during implementation was widely addressed [22,23,24,35]. A Dutch evaluation of the implementation of the Transmural Trauma Care Model found that patients' experience improved as more focus was placed on day-to-day issues patients encountered [22]. However, time constraints were a key barrier to providing improved patient care [10,16,22,24, 31,32,34,37,]. A Peruvian study on the status of in-hospital TQIPs revealed the *"prevalence of dual practice"* [10 p.966], surgeons working at both public and private institutions, meant that clinicians did not have time to effectively participate in emerging quality improvement initiatives.

Collaboration with national and international bodies was a facilitator to successful implementation [17,23]. A questionnaire with trauma registry stewards in LMICs showed a need to *"develop skills at political lobbying to induce support from the administration*" to provide a platform for long-term aid [17 p.2221]. The lack of common guidelines was highlighted as a barrier [10,17,21,28, 32,38]. A study exploring staff trauma experiences in LMICs underlined that organisational diversity across the region led to limited uniform policies which reduced communication among trauma facilities [10].

For peer review only - http://bmjopen.bmj.com/site/about/guidelines.xhtml

Domain 3: Inner setting

Increased staff education and training strengthened the implementation climate of quality improvement interventions [18,19,20,21,24,25,29,35,36,39,41]. An integrated self-management support paper emphasised successful training as accessible to staff at all levels, with a focus on flexibility [24]. Professional hierarchies acted as a barrier to the continued education of all staff [10,19,24,25]. A training program clinician survey found that traditional decision-making hierarchies acted as a major barrier to effective education as team members were *"reluctant to speak up to highlight a problem, clarify information, or question a senior's decision when they were concerned"* [25 p.1151].

Implementation readiness was positively impacted by prioritising leadership buy-in [10,17,20,35,39]. An electronic registry in Pakistan highlighted obtaining senior management buy-in as a catalyst to improving trauma care quality [39]. Lack of stakeholder buy-in acted as a barrier to achieving progress in trauma care [17,21]. A trauma registry custodian from an LMIC hospital stated that a common opinion was that *"even basic care is not being given to the patients…how are we going to sort of spend for a registry?"* [21 p.122].

Diversifying resource sources is fundamental for successful implementation [17,29,42]. Respondents in a resource-constrained setting highlighted demand should be met by broadening the registry scope to be eligible for more grants [17]. This was in response to a common barrier around limited resources [17,18,21,23,29,36,39,40,42]. A trauma registry custodian explained that staff must *"make do with the resources that you have because the funding will not be there"* [23 p.120].

Domain 4: Characteristics of individuals

Generating staff understanding of quality improvement is needed to encourage change [10,20,21]. Increased quality improvement dissemination can help develop greater public awareness [10,20]. A barrier to this effort is the lack of staff belief in quality improvement interventions [10,16,17,30]. A participant from a healthcare provider interview on implementing the WHOTCC highlighted

For peer review only - http://bmjopen.bmj.com/site/about/guidelines.xhtml

that *"nobody is really trying to do it as a career"* as the majority *"would rather be doing joint replacements, rather than worrying about the poor people falling off trains and motorcycles"* [30 p.19].

Communication training was a facilitator for TQIP implementation [16,24,25,31,32,34]. A trauma training clinician survey emphasised developing interpersonal staff communication alongside strengthening emergency communication methods [32]. Limited staff knowledge acts as a hurdle to improved communication [10,26,31,34]. A participant from a US injury prevention program highlighted the lack of healthy understanding of quality improvement requirements as they are viewed as *"the black sheep"* of the department, making it a constant struggle to *"seek out other mentors of people that do similar work"* [26 p.339].

Domain 5: Process of implementation

Identifying specific individuals in each organisation to drive the quality improvement intervention as a champion is significant to improve prioritisation [17,26,30,36]. Trauma registry questionnaire respondents felt that limited stakeholder engagement could be overcome by having a champion to drive and mobilise buy-in [17]. Identifying a champion is made difficult by the barrier of overworked staff [10,16,18,31,17,34,37]. A Tanzanian hospital was so understaffed that *"you will find you have one doctor at night and ten patients show up at once"*, making it difficult to document records and motivate staff [37 p.26].

Strengthening data quality was highlighted as a facilitator [16,19,20,21,39,40,41,42]. Asia-Pacific trauma leaders proposed establishing standardised minimum data requirements to collect more complete patient information and integrate injury surveillance with data collection [19]. These efforts were hindered by poor data quality across country income levels [17,18,19,21,23,32,41,42]. An Argentinian registry implementation project found a large variation in trauma admission protocols and widespread under-reporting, impacting the quality of clinician records [32].

Common facilitators and barriers were arranged by income level and are summarised in table 3.

For peer review only - http://bmjopen.bmj.com/site/about/guidelines.xhtml

Table 3. Main facilitators and barriers to TQIP implementation, by income level

Income Level	Facilitators	Barriers
L	• Staff education & training<br>• Open dialogue with key stakeholders<br>• Strengthening data quality	• Poor Data Quality<br>• Limited Resources<br>• Overworked Staff
LM	• Staff education & training<br>• Tailoring to local context<br>• Strengthening data quality	• Poor data quality<br>• Lack of buy-in<br>• Lack of common guidelines
UM	• Staff education & training<br>• Tailoring to local context<br>• Prioritising buy-in	• Poor data quality<br>• Lack of common guidelines<br>• Lack of staff knowledge
H	• Staff education & training<br>• Communication training<br>• Prioritising patient care	• Professional hierarchies<br>• Lack of common guidelines<br>• Overworked staff

Discussion

In-hospital TQIP facilitators and barriers were similar across all income levels. Facilitators and barriers, grouped under the five CFIR domains, identified prioritising staff education and training, strengthening dialogue with stakeholders, and increasing standardised guidelines for best practice as key facilitators going forward. Major barriers were the need to prioritise data quality improvement in LMICs and improved communication training in HICs. Studies focusing on in-hospital TQIP research in LMICs were limited to a few countries, indicating that the geographic scope of quality improvement research must be widened in these regions.

A facilitator was strengthening stakeholder dialogue to increase buy-in for in-hospital TQIPs. This supports a previous qualitative interview study that proposed adopting a hybrid quality

For peer review only - http://bmjopen.bmj.com/site/about/guidelines.xhtml

improvement model where experts would set basic guidelines for programs, incorporating feedback from frontline staff members according to local conditions [43]. Key barriers to stakeholder dialogue included a limited desire for TQIPs, resistance to change, and competing group interests. Studies reported the need to tailor interventions to the local context, along with focusing on simplicity to improve communication. An earlier grounded theory analysis of HIC interviews identified important strategies to improve stakeholder engagement as clarity of purpose, inviting participation, and engaging clinicians with feedback [44]. Given the wide range of competing innovations in hospital settings, focusing on increasing buy-in through stakeholder dialogue could strengthen the likelihood of effective implementation along with positive patient outcomes.

A reported barrier across all country income levels was the lack of common guidelines throughout the trauma care timeline. A review of LMIC trauma registry implementation highlighted that the WHO's *Injury Surveillance Guidelines* are only focused on collecting data on injury events rather than also addressing hospital care or trauma outcomes [45]. The uncoordinated and resource-intensive nature of data collection acted as a barrier that contributed to staff time constraints. A survey revealed that there is no single resource available that provides a comprehensive insight into experiences across trauma registries [46]. To overcome this, quality improvement guideline standardisation was emphasised as a facilitator to improve the success of in-hospital implementation.

Limited in-hospital TQIP durability data underlines the need to prioritise research on sustainable interventions. An LMIC scoping review on trauma registry implementation highlighted the need to prioritise sustainable registry development as a significant step forward [45]. Key barriers reported were the lack of long-term funding and limited buy-in for quality improvement initiatives. In our review, financial resources were not reported as widely as initially expected, particularly in LMICs. Despite this, key identified barriers such as staff time constraints and limited resources are directly linked to a lack of funding. Increased dissemination of results is a critical facilitator to establish prolonged TQIP support, through enhancing engagement and quality improvement understanding. A trauma care provider survey in the Andean region concluded that increased quality improvement dissemination can empower local providers to participate in health systems and drive change [37]. The increase in peer-reviewed quality improvement reports from LMICs

For peer review only - http://bmjopen.bmj.com/site/about/guidelines.xhtml

emphasises dissemination through publications as a viable means of providing guidance for future programs and expanding quality improvement knowledge [47].

Education and training are essential facilitators to improve the chances of successful implementation. This was mentioned at all country income levels, specifically in terms of interpersonal communication in HICs. Enhancing communication channels between trauma teams to improve health outcomes is increasingly common in HICs. A quantitative study found that well-defined interdisciplinary communication between the trauma service and surgical specialists reduced the time-to-operation for facial trauma patients [48]. Common barriers were limited staff belief and knowledge in quality improvement interventions. While elements of the WHO's guidelines for TQIPs have been mandated by governments across income levels [49], there have not been clear protocols put into place for implementing, evaluating, and sustaining interventions. This makes it difficult to provide standardised training for staff, which can impact the quality of patient care.

Enhancing data quality is a crucial facilitator to strengthening in-hospital implementation. Trauma registries were the most common in-hospital TQIP intervention, particularly in LMICs, with studies citing poor data quality as the main barrier. Reasons for the lack of data quality centered on high rates of missing data and limited standardised variables for comparison. A scoping review assessing trauma registry data quality found that only four out of sixty-nine publications provided a general classification of data quality, in which the taxonomy was inconsistent [50]. A similar systematic review identified only ten studies that evaluated data quality in trauma registries [51]. The majority assessed data quality based on completeness, with large differences reported between papers, and no evidence found on data precision or timeliness [51]. This lack of uniformity makes it difficult to draw insights on the quality of trauma care delivered and compare across country income levels. The Lancet Global Health Commission on High Quality Health Systems underlined the disparity between the growing global injury burden and the limited data availability on care quality [52]. Providing incentives for data entry participation, such as hospital-specific dashboards or research involvement, could improve data quality and support effective in-hospital implementation.

For peer review only - http://bmjopen.bmj.com/site/about/guidelines.xhtml

Strengths and limitations

The review covered a broad range of in-hospital TQIP interventions and included studies with any design. The screening process was conducted along with two peer reviewers, which allowed for comparison and greater input in decision-making – increasing protection against potential bias This review provides a snapshot of the research field through a methodologically robust process which includes a transparent method of reviewing in a limited timeframe [53]. The review captured a large number of studies for analysis from various databases.

The use of three databases and the restriction of literature to English-language studies could have biased the findings if excluded studies were significantly different to those included in the scoping review. The use of more databases, including preprint servers, a wider timeframe, and the inclusion of a greater number of studies could have provided data that may have been neglected. We attempted to limit the selection bias by consulting a librarian with extensive experience conducting scoping reviews when selecting databases.

Grouping literature into income levels by country may provide another limitation. This classification does not capture the complexity of resource allocation within countries, reducing the generalisability of the results. The scoping review process does not place as much emphasis on assessing the quality of included studies when compared with systematic reviews [54]. The lack of quality assessment limits the implications of gathered data as the study focus is primarily concerned with mapping available literature on the topic. There is a risk of researcher bias as the process of choosing and analysing literature can be subjective. Important steps to avoid researcher bias were focusing on the research question, following the eligibility criteria, and cross-referencing screening selection with a peer–reviewer.

Conclusion

Staff education and training, strengthening stakeholder dialogue, and increasing standardised trauma care guidelines were reported as the main facilitators, while major barriers identified were poor data quality in LMICs and the lack of effective communication training in HICs. Stakeholder

For peer review only - http://bmjopen.bmj.com/site/about/guidelines.xhtml

prioritisation of TQIPs, along with increased knowledge and consensus on trauma care best practice could further advance efforts to lower the global trauma burden. The focus of future in-hospital TQIPs in LMICs should primarily be concerned with improving the data quality of registries, while interventions in HICs should focus on the communication skills of healthcare professionals.

For pe

*** ***

Acknowledgements

We would like to acknowledge Karolinska Institutet Library (Independent Information Specialist Consultants, Solna, Sweden) for peer review of the literature search strategy, along with the faculty and students of Karolinska Institutet Department of Global Public Health for comments and feedback on earlier versions of this manuscript.

Funding

None.

Data availability statement

The data that support the findings of this study are available from PubMed, Web of Science, and Global Index Medicus databases using the search string outlined in Online Supplement 1.

Contributors

Study concept, design and search strategy: GK, JB, MGW. Data selection and screening: GK, JB, YS, MGW. Data analysis and interpretation: GK, JB, MGW. Drafting of manuscript: GK, JB, MGW. Critical revisions of manuscript: GK, JB, YS, MGW. Study supervision: JB, MGW. All authors read and approved the final manuscript.

For peer review only - http://bmjopen.bmj.com/site/about/guidelines.xhtml

Ethics approval and consent to participate

Not applicable.

Consent for publication

Not applicable

Competing interests

The authors declare that they have no competing interests.

References

1. Gerdin M. The risk of dying: predicting trauma mortality in urban Indian hospitals [dissertation on the internet]. Stockholm: Karolinska Institutet; 2015 [cited 2022 Mar 23]. Available from: https://openarchive.ki.se/xmlui/bitstream/handle/10616/44832/Thesis_Martin_Gerdin.pdf
2. Vos T, Lim SS, Abbafati C, Abbas KM, Abbasi M, Abbasifard M, et al. Global burden of 369 diseases and injuries in 204 countries and territories, 1990–2019: a systematic analysis for the Global Burden of Disease Study 2019. Lancet. 2020 Oct 17;396(10258):1204-22.
3. Mock C, Juillard C, Brundage S, Goosen J, Joshipura M. Guidelines for trauma quality improvement programmes. Geneva: World Health Organization, 2009.
4. Cole E, Lecky F, West A, Smith N, Brohi K, Davenport R, et al. The impact of a pan-regional inclusive trauma system on quality of care. Ann Surg. 2016; 264(1):188–94.
5. Itri JN, Bakow E, Probyn L, Kadom N, Duong PA, Gettle LM, et al. The science of quality improvement. Acad Radiol. 2017 Mar;24(3):253-62.
6. Pope C, Van Royen P, Baker R. Qualitative methods in research on healthcare quality. Qual Saf Health Care. 2002 Jun;11(2):148-52.

For peer review only - http://bmjopen.bmj.com/site/about/guidelines.xhtml

7. Alexander JA, Weiner BJ, Shortell SM, Baker LC, Becker MP. The role of organizational infrastructure in implementation of hospitals' quality improvement. Hosp Top. Winter 2006;84(1):11-21.
8. Alexander JA, Hearld LR. The science of quality improvement implementation: developing capacity to make a difference. Med care. 2011 Dec; 49 Suppl:S6-20.
9. Reynolds TA, Stewart B, Drewett I, Salerno S, Sawe HR, Toroyan T, et al. The impact of trauma care systems in low-and middle-income countries. Annu Rev Public Health. 2017 Mar 20;38:507-32.
10. LaGrone LN, Fuhs AK, Egoavil EH, Rodriguez Castro MJ, Valderrama R, IsquithDicker LN, et al. Mixed-methods assessment of trauma and acute care surgical quality improvement programs in Peru. World J Surg. 2017 Apr;41(4):963-969.
11. Tricco AC, Lillie E, Zarin W, O'Brien KK, Colquhoun H, Levac D,et alS. PRISMA extension for scoping reviews (PRISMA-ScR): checklist and explanation. Ann Intern Med. 2018 Oct 2;169(7):467-473.
12. WDI - The World by Income and Region [Internet]. Datatopics.worldbank.org. 2022 [cited 2022 April 25]. Available from: https://datatopics.worldbank.org/world-development-indicators/the-world-by-income-and-region.
13. Damschroder LJ, Aron DC, Keith RE, Kirsh SR, Alexander JA, Lowery JC. Fostering implementation of health services research findings into practice: a consolidated framework for advancing implementation science. Implement Sci. 2009 Aug 7; 4:50.
14. CFIR Research Team-Center for Clinical Management Research. Consolidated Framework for Implementation Research [Internet]. Ann Arbor: CFIR Research Team-Center for Clinical Management Research; 2022. [cited 2022 April 12]. Available from: https://cfirguide.org/
15. Damschroder LJ, Lowery JC. Evaluation of a large-scale weight management program using the consolidated framework for implementation research (CFIR). Implement Sci. 2013 May 10; 8:51.
16. Croke K, Chokotho L, Milusheva S, Bertfelt J, Karpe S, Mohammed M, et al. Implementation of a multi-center digital trauma registry: Experience in district and central hospitals in Malawi. Int J Health Plann Manage. 2020 Sep;35(5):1157-1172.

For peer review only

17. Rosenkrantz L, Schuurman N, Arenas C, Jimenez MF, Hameed MS. Understanding the barriers and facilitators to trauma registry development in resource-constrained settings: a survey of trauma registry stewards and researchers. Injury. 2021 Aug;52(8):2215-2224.

18. Hamadani F, Razek T, Massinga E, Gupta S, Muataco M, Muripiha P, et al. Trauma surveillance and registry development in Mozambique: results of a 1-year study and the first phase of national implementation. World J Surg. 2019 Jul;43(7):1628-1635.

19. Stelfox HT, Joshipura M, Chadbunchachai W, Ellawala RN, O'Reilly G, Nguyen TS, et al. Trauma quality improvement in low and middle income countries of the Asia– Pacific region: a mixed methods study. World J Surg. 2012 Aug;36(8):1978-1992.

20. Stevens KA, Paruk F, Bachani AM, Wesson HH, Wekesa JM, Mburu J,et al. Establishing hospital-based trauma registry systems: lessons from Kenya. Injury. 2013 Dec;44 (Suppl 4): S70-S74.

21. O'Reilly GM, Gabbe B, Braaf S, Cameron PA. An interview of trauma registry custodians to determine lessons learnt. Injury. 2016 Jan;47(1):116-1.

22. Wiertsema SH, Donker MH, van Dongen JM, Geleijn E, Bloemers FW, Ostelo RW, et al. The Transmural Trauma Care Model can be implemented well but some barriers 46 and facilitators should be considered during implementation: a mixed methods study. J Physiother. 2021 Oct;67(4):298-307.

23. Hashmi ZG, Haider AH, Zafar SN, Kisat M, Moosa A, Siddiqui F, et al. Hospitalbased trauma quality improvement initiatives: first step toward improving trauma outcomes in the developing world. J Trauma Acute Care Surg. 2013 Jul;75(1):60-68.

24. Hollinshead L, Jones F, Silvester L, Marshall-Taylor P. Implementing an integrated approach to self-management support in an acute major trauma therapy team: an improvement project. BMJ Open Qual. 2019 Jul 4;8(3): e000415.

25. Murphy M, Curtis K, McCloughen A. Facilitators and barriers to the clinical application of teamwork skills taught in multidisciplinary simulated trauma team training. Injury. 2019 May;50(5):1147-1152.

26. Newcomb AB, Zadnik M, Carlini AR, Francis MM, Frey KP, Heins SE, et al. Barriers and facilitators to the implementation of injury prevention programs: a qualitative exploration and model development. J Trauma Nurs. 2020;27(6):335-345.

For peer review only - http://bmjopen.bmj.com/site/about/guidelines.xhtml

27. LaGrone LN, Pozo DA, Figueroa JF, Artunduaga MA, Egoavil EH, Castro MJ, et al. Status of trauma quality improvement programs in the Andean region: what foundation do we have to build on. Injury. 2017 Sep;48(9):1985-1993.

28. Rosenkrantz L, Schuurman N, Arenas C, Nicol A, Hameed MS. Maximizing the potential of trauma registries in low-income and middle-income countries. Trauma Surg Acute Care Open. 2020 May 12;5(1): e000469.

29. Parreira JG, Campos TD, Perlingeiro JA, Soldá SC, Assef JC, Gonçalves AC, et al. Implementation of the trauma registry as a tool for quality improvement in trauma care in a Brazilian hospital: the first 12 months. Rev Col Bras Cir. 2015 Jul;42(4):265- 272.

30. Wild H, Mock C, Lim A. Implementation of the WHO Trauma Care Checklist: A qualitative analysis of facilitators and barriers to use. Int J Surg. 2020 Nov; 83:15-23.

31. Fuangworawong P, LaGrone LN, Chadbunchachai W, Sornsrivichai V, Mock CN, Vavilala MS. Assessment of trauma quality improvement activities at public hospitals in Thailand. Int J Surg. 2016 Sep;33 Pt A:88-95.

32. Monteverde E, Santero M, Bosque L, Lartigue B, Tenaillon C, Baliña J, et al. A public–private collaborative model for a trauma program implementation: findings from a prospective trauma registry at 14 hospitals in Buenos Aires, Argentina. Eur J Trauma Emerg Surg. 2021 Dec;47(6):1931-1937.

33. Schuurman N, Cinnamon J, Matzopoulos R, Fawcett V, Nicol A, Hameed SM. Collecting injury surveillance data in low-and middle-income countries: The Cape Town Trauma Registry pilot. Glob Public Health. 2011;6(8):874-889.

34. Sawe HR, Sirili N, Weber E, Coats TJ, Reynolds TA, Wallis LA. Perceptions of health providers towards the use of standardised trauma form in managing trauma patients: a qualitative study from Tanzania. Inj Epidemiol. 2020 May 1;7(1):15.

35. Wainiqolo I, Kafoa B, McCaig E, Kool B, McIntyre R, Ameratunga S. Development and piloting of the Fiji injury surveillance in hospitals system (TRIP Project-1). Injury. 2013 Jan;44(1):126-131.

36. Zargaran E, Schuurman N, Nicol AJ, Matzopoulos R, Cinnamon J, Taulu T, et al. The electronic Trauma Health Record: design and usability of a novel tablet-based tool for trauma care and injury surveillance in low resource settings. J Am Coll Surg. 2014 Jan;218(1):41-50.

For peer review only - http://bmjopen.bmj.com/site/about/guidelines.xhtml

37. Sawe HR, Sirili N, Weber E, Coats TJ, Wallis LA, Reynolds TA. Barriers and facilitators to implementing trauma registries in low-and middle-income countries: qualitative experiences from Tanzania. Afr J Emerg Med. 2020;10(Suppl 1): S23-S28.

38. Zhou J, Wang T, Belenkiy I, Hardcastle TC, Rouby JJ, Jiang B. Management of severe trauma worldwide: implementation of trauma systems in emerging countries: China, Russia and South Africa. Crit Care. 2021 Aug 9;25(1):286.

39. Mehmood A, Razzak JA, Kabir S, MacKenzie EJ, Hyder AA. Development and pilot implementation of a locally developed Trauma Registry: lessons learnt in a low-income country. BMC Emerg Med. 2013 Dec 21;13:4.

40. St-Louis E, Paradis T, Landry T, Poenaru D. Factors contributing to successful trauma registry implementation in low-and middle-income countries: a systematic review. Injury. 2018 Dec;49(12):2100-2110.

41. Chokotho LC, Mulwafu W, Nyirenda M, Mbomuwa FJ, Pandit HG, Le G, et al. Establishment of trauma registry at Queen Elizabeth Central Hospital (QECH), Blantyre, Malawi and mapping of high-risk geographic areas for trauma. World J Emerg Med. 2019;10(1):33-41.

42. Bommakanti K, Feldhaus I, Motwani G, Dicker RA, Juillard C. Trauma registry implementation in low-and middle-income countries: challenges and opportunities. J Surg Res. 2018 Mar; 223:72-86.

43. Parker LE, Kirchner JE, Bonner LM, Fickel JJ, Ritchie MJ, Simons CE, et al. Creating a quality-improvement dialogue: utilising knowledge from frontline staff, managers, and experts to foster health care quality improvement. Qual Health Res. 2009 Feb;19(2):229-242

44. Santana MJ, Straus S, Gruen R, Stelfox HT. A qualitative study to identify opportunities for improving trauma quality improvement. J Crit Care. 2012 Dec;27(6):738.e1-7.

45. Rosenkrantz L, Schuurman N, Hameed M. Trauma registry implementation and operation in low and middle income countries: a scoping review. Glob Public Health. 2019 Dec;14(12):1884-1897.

46. O'Reilly GM, Gabbe B, Cameron PA. Trauma registry methodology: a survey of trauma registry custodians to determine current approaches. Injury. 2015 Feb;46(2):201-206.

For peer review only - http://bmjopen.bmj.com/site/about/guidelines.xhtml

47. Sifrim ZK, Barker PM, Mate KS. What gets published: the characteristics of quality improvement research articles from low-and middle-income countries. BMJ Qual Saf. 2012 May;21(5):423-431
48. Keller PR, Schneeberger S, Drolet BC, Al Kassis S, Pearce FB, Perdikis G. Implementation of a trauma quality improvement program communications package decreases time-to-operation for facial trauma. Ann Plast Surg. 2019 Jun;82(6S Suppl 5):S380-S385.
49. Bardfield J, Agins B, Akiyama M, Basenero A, Luphala P, Kaindjee-Tjituka F, et al. A quality improvement approach to capacity building in low-and middle-income countries. Aids. 2015 Jul;29(Suppl 2):S179-S186.
50. O'Reilly GM, Gabbe B, Moore L, Cameron PA. Classifying, measuring and improving the quality of data in trauma registries: a review of the literature. Injury. 2016 Mar;47(3):559-567.
51. Porgo TV, Moore L, Tardif PA. Evidence of data quality in trauma registries: a systematic review. J Trauma Acute Care Surg. 2016 Apr;80(4):648-658.
52. Whitaker J, Nepogodiev D, Leather A, Davies J. Assessing barriers to quality trauma care in low and middle-income countries: a Delphi study. Injury. 2020 Feb;51(2):278- 285
53. Gosselin RA, Spiegel DA, Coughlin R, Zirkle LG. Injuries: the neglected burden in developing countries. Bull World Health Organ. 2009 Apr;87(4):246-246a.
54. Arksey H, O'Malley L. Scoping studies: towards a methodological framework. Int J Soc Res Methodol. 2005 Feb;8(1):19-32.

Figure titles/legends:

Figure 1: CFIR conceptual framework outline

Adapted from Damschroder et al, 2009 [13].

Figure 2: Study selection

For peer review only - http://bmjopen.bmj.com/site/about/guidelines.xhtml

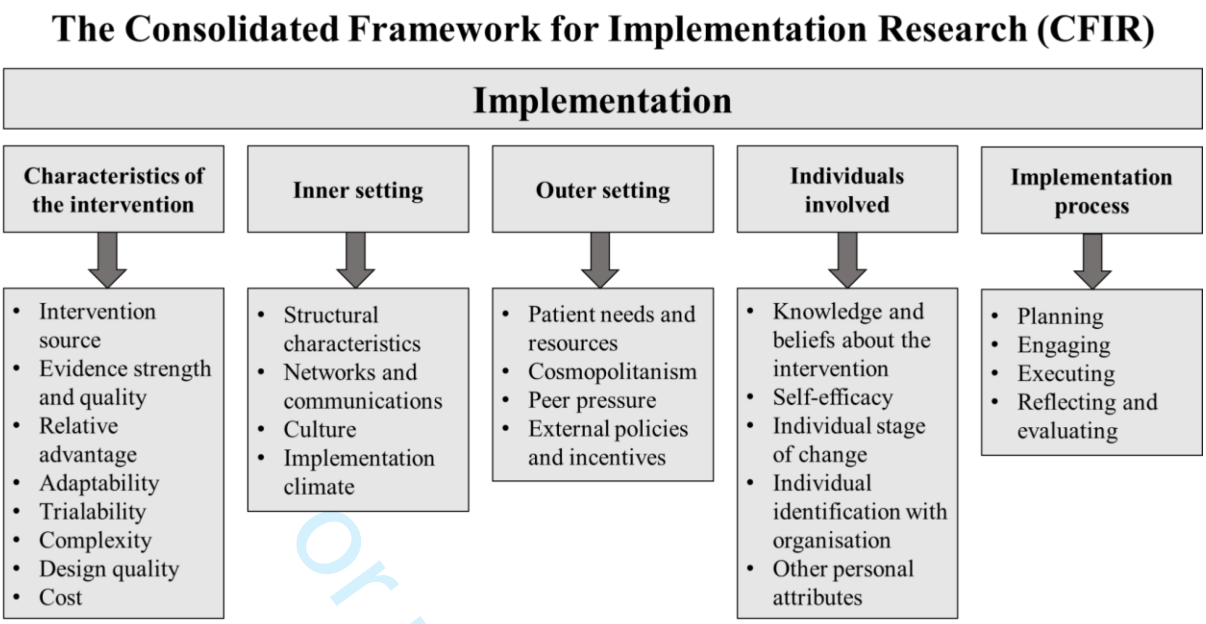

For peer review only

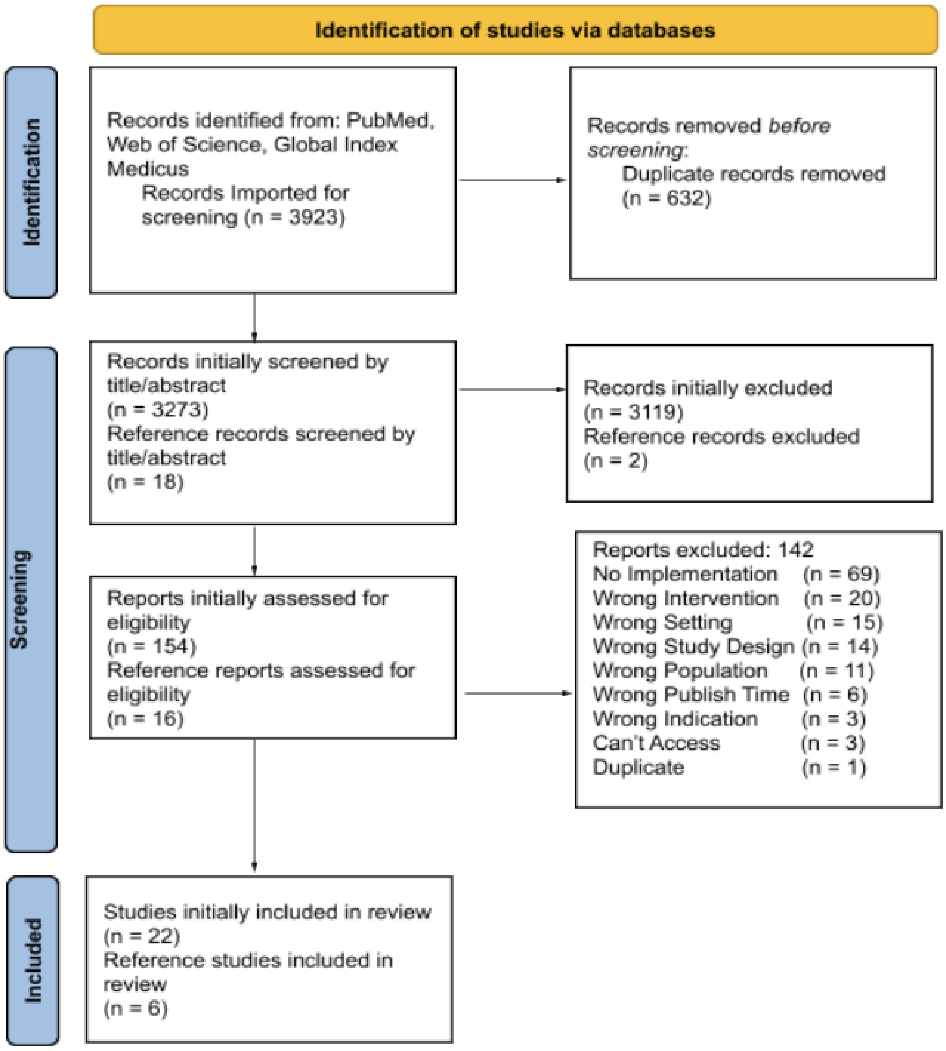
Identification of studies via databases
Identification
Screening
Included
Records identified from: PubMed, Web of Science, Global Index Medicus
Records Imported for screening (n = 3923)
Records removed *before screening*:
Duplicate records removed (n = 632)
Records initially screened by title/abstract (n = 3273)
Reference records screened by title/abstract (n = 18)
Records initially excluded (n = 3119)
Reference records excluded (n = 2)
Reports initially assessed for eligibility (n = 154)
Reference reports assessed for eligibility (n = 16)
Reports excluded: 142
No Implementation (n = 69)
Wrong Intervention (n = 20)
Wrong Setting (n = 15)
Wrong Study Design (n = 14)
Wrong Population (n = 11)
Wrong Publish Time (n = 6)
Wrong Indication (n = 3)
Can't Access (n = 3)
Duplicate (n = 1)
Studies initially included in review (n = 22)
Reference studies included in review (n = 6)

Online Supplement:

Online Supplement 1: Database search strategy according to Population, Concept, Context (PCC) framework

**Population:**		
**#1**	**MeSH terms:**	Humans and Trauma Centres
**#2**	**Free Text:**	Population OR populations OR patient OR patients OR adult OR adults OR injured OR victim OR victims OR trauma centre OR trauma centres OR trauma department OR trauma departments OR trauma facility OR trauma facilities OR acute care centre OR acute care centres OR acute care department OR acute care departments OR acute care facility OR acute care facilities OR primary care centre OR primary care centres OR primary care department OR primary care departments OR primary care facility OR primary facilities OR emergency centre OR emergency centres OR emergency department OR emergency departments OR emergency facility OR emergency facilities
**#3**	**#1 OR #2**	
**Concept:**		
**#4**	**MeSH terms:**	Hospital and quality improvement
**#5**	**Free Text:**	Hospital OR hospitals OR in-hospital OR hospital-based OR facility OR facilities AND quality improvement OR quality indicator OR facilitator OR facilitators OR opportunity OR opportunities OR advantage OR advantages OR solution OR solutions OR barrier OR barriers OR challenge OR challenges OR disadvantage OR disadvantages OR inhibitor OR inhibitors OR implementation OR intervention OR innovation
**#6**	**#4 OR #5**	
**Context:**		
**#7**	**MeSH terms:**	Developing countries and developed countries
**#8**	**Free Text:**	Developing country OR developing countries OR developing nation OR developing nations OR developing economy OR developing economies OR low income country OR low income countries OR low income nation OR low income nations OR low income economy OR low income economies OR lower middle income

		country OR lower middle income countries OR lower middle income nation OR lower middle income nations OR lower middle income economy OR lower middle income economies OR upper middle income country OR upper middle income countries OR upper middle income nation OR upper middle income nations OR upper middle income economy OR upper middle income economies OR middle income country OR middle income countries OR middle income nation OR middle income nations OR middle income economy OR middle income economies OR transitional country OR transitional countries OR transitional nation OR transitional nations OR transitional economy OR transitional economies OR developed country OR developed countries OR developed nation OR developed nations OR developed economy OR developed economies OR high income country OR high income countries OR high income nation OR high income nations OR high income economy OR high income economies
**#9**	**#7 OR #8**	
**#10**	**#3 AND #6 OR #9**	3923 Results
**Filter**	English language studies published from June 2009 onwards	

Online Supplement 2: Characteristics of extracted studies

Income Level	Continents/Countries	Source	Study Type	Intervention Type	Intervention Duration (months)
L	Malawi	Chokotho et al. 2019 [16]	Qualitative - Survey	T.R	N/A
L	Malawi	Croke et al. 2020 [17]	Qualitative - Survey	T.R	N/A
L	Mozambique	Hamadani et al. 2019 [18]	Qualitative - Interview	T.R	12
L	Tanzania[a]	Sawe et al. 2020 [19]	Qualitative - Focus Group Discussion	T.R	N/A
L	Tanzania[a]	Sawe et al. 2020 [20]	Qualitative - Interview	T.R	N/A
LM	Pakistan	Hashmi et al. 2013 [21]	Quantitative - logistic regression	T.R, Q.A.P	108
LM	Pakistan	Mehmood et al. 2013 [22]	Qualitative - Survey	T.R	3

For peer review only

LM	Asia: 7	Stelfox et al. 2012 [23]	Mixed Methods	Range - M&M, PDP, AF, QOC Audit, Statistical Analysis	N/A
LM	Kenya	Stevens et al. [24]	Qualitative - Survey	T.R	N/A
UM	Thailand	Fuangworawong et al. 2016 [25]	Qualitative - Survey	Range - M&M, T.R, A.F, PDP	N/A
UM	Peru	LaGrone et al. 2017 [10]	Mixed methods	Range - M&M/T.R	N/A
UM	Argentina	Monteverde et al. 2021 [26]	Qualitative - Univariate/Bivariate descriptive analysis	T.R	114
UM	Brazil	Parreira et al. 2015 [27]	Qualitative - Survey	T.R	12
UM	South Africa	Schuurman et al. 2011 [28]	Qualitative - Survey	T.R	1
UM	Fiji	Wainiqolo et al. 2013 [29]	Qualitative - Survey	T.R	5
UM	Africa: 1, Asia: 1, Europe: 1	Zhou et al. 2021 [30]	Literature Review	T.R	N/A
H	U.K	Hollinshead et al. 2019 [31]	Qualitative - Survey	Educational - B.S.M.P	12
H	Australia	Murphy et al. 2019 [32]	Qualitative - Survey	Multidisciplinary TTT	N/A
H	U.S.A	Newcomb et al. 2020 [33]	Qualitative - Interview	I.P.P	N/A
H	Netherlands	Wiertsema et al. 2021 [34]	Mixed Methods	TTCM	N/A
Range - LMIC	Africa: 6, Asia: 3, South America: 3, North America: 1	Rosenkrantz et al. 2021 [35]	Qualitative - Survey	T.R	Range - 3 = <24 1 = 24-60, 7 = 72-120, 2 = >120
Range - LMIC	Africa: 9, Asia: 4, South	St-Louis et al. 2018 [36]	Literature Review	T.R	N/A

For peer review only

	America: 3, North America: 1, Oceania: 1				
Range - LM/UM	South America: 4	LaGrone et al. 2017 [37]	Qualitative - Survey	Range - M&M, T.R, A.F, Autopsies	N/A
Range - UM/H	South Africa/Canada	Zargaran et al. 2014 [38]	Qualitative - Survey	T.R	1
Range - All	Africa: 6, Asia: 6, North America: 3, South America: 2, Europe: 1, Oceania: 1	Bommakanti et al. 2018 [39]	Literature Review	T.R	N/A
Range - All	Africa: 8, Asia: 6, Europe: 3, North America: 3, Oceania: 2	O'Reilly et al. 2016 [40]	Qualitative - Interview	T.R	N/A
Range - All	Asia: 5, Africa: 3, South America: 1, North America: 1, Oceania: 1	Wild et al. 2020 [41]	Qualitative - Interview	TCC	24
Range - Unspecified	Africa: 7, Asia: 3, North America: 2, Europe: 1, Oceania: 1	Rosenkrantz et al. 2020 [42]	Literature Review	T.R	N/A

Note. L = Low Income, LM = Lower Middle Income, UM = Upper Middle Income, H = High Income, T.R = Trauma Registry, Q.A.P = Quality Assurance Program, M&M = Mortality & Morbidity Conferences, PDP = Preventable Death Panel, A.F = Audit Filter, QOC = Quality of Care, B.S.M.P = Bridges Self-Management Programme, TTT = Trauma Team Training, I.P.P = Injury Prevention Program, TTCM = Transmural Trauma Care Model, TCC = Trauma Care Checklist.

"Qualitative - Survey" design included studies that conducted questionnaires

[a]Tanzania reclassified from low to lower middle income country after data collection of studies

For peer review only

Preferred Reporting Items for Systematic reviews and Meta-Analyses extension for Scoping Reviews (PRISMA-ScR) Checklist

SECTION	ITEM	PRISMA-ScR CHECKLIST ITEM	REPORTED ON PAGE #
**TITLE**			
Title	1	Identify the report as a scoping review.	1
**ABSTRACT**			
Structured summary	2	Provide a structured summary that includes (as applicable): background, objectives, eligibility criteria, sources of evidence, charting methods, results, and conclusions that relate to the review questions and objectives.	2
**INTRODUCTION**			
Rationale	3	Describe the rationale for the review in the context of what is already known. Explain why the review questions/objectives lend themselves to a scoping review approach.	4
Objectives	4	Provide an explicit statement of the questions and objectives being addressed with reference to their key elements (e.g., population or participants, concepts, and context) or other relevant key elements used to conceptualize the review questions and/or objectives.	4
**METHODS**			
Protocol and registration	5	Indicate whether a review protocol exists; state if and where it can be accessed (e.g., a Web address); and if available, provide registration information, including the registration number.	5
Eligibility criteria	6	Specify characteristics of the sources of evidence used as eligibility criteria (e.g., years considered, language, and publication status), and provide a rationale.	5
Information sources*	7	Describe all information sources in the search (e.g., databases with dates of coverage and contact with authors to identify additional sources), as well as the date the most recent search was executed.	5
Search	8	Present the full electronic search strategy for at least 1 database, including any limits used, such that it could be repeated.	Online Supplement 1
Selection of sources of evidence†	9	State the process for selecting sources of evidence (i.e., screening and eligibility) included in the scoping review.	5
Data charting process‡	10	Describe the methods of charting data from the included sources of evidence (e.g., calibrated forms or forms that have been tested by the team before their use, and whether data charting was done independently or in duplicate) and any processes for obtaining and confirming data from investigators.	5
Data items	11	List and define all variables for which data were sought and any assumptions and simplifications made.	5
Critical appraisal of individual sources of evidence§	12	If done, provide a rationale for conducting a critical appraisal of included sources of evidence; describe the methods used and how this information was used in any data synthesis (if appropriate).	N/A

St. Michael's
Inspired Care.
Inspiring Science.

For peer review only - http://bmjopen.bmj.com/site/about/guidelines.xhtml

SECTION	ITEM	PRISMA-ScR CHECKLIST ITEM	REPORTED ON PAGE #
Synthesis of results	13	Describe the methods of handling and summarizing the data that were charted.	6
**RESULTS**			
Selection of sources of evidence	14	Give numbers of sources of evidence screened, assessed for eligibility, and included in the review, with reasons for exclusions at each stage, ideally using a flow diagram.	7
Characteristics of sources of evidence	15	For each source of evidence, present characteristics for which data were charted and provide the citations.	Online Supplement 2
Critical appraisal within sources of evidence	16	If done, present data on critical appraisal of included sources of evidence (see item 12).	N/A
Results of individual sources of evidence	17	For each included source of evidence, present the relevant data that were charted that relate to the review questions and objectives.	7-15
Synthesis of results	18	Summarize and/or present the charting results as they relate to the review questions and objectives.	15
**DISCUSSION**			
Summary of evidence	19	Summarize the main results (including an overview of concepts, themes, and types of evidence available), link to the review questions and objectives, and consider the relevance to key groups.	16-18
Limitations	20	Discuss the limitations of the scoping review process.	18
Conclusions	21	Provide a general interpretation of the results with respect to the review questions and objectives, as well as potential implications and/or next steps.	18-19
**FUNDING**			
Funding	22	Describe sources of funding for the included sources of evidence, as well as sources of funding for the scoping review. Describe the role of the funders of the scoping review.	25

JBI = Joanna Briggs Institute; PRISMA-ScR = Preferred Reporting Items for Systematic reviews and Meta-Analyses extension for Scoping Reviews.

* Where *sources of evidence* (see second footnote) are compiled from, such as bibliographic databases, social media platforms, and Web sites.

† A more inclusive/heterogeneous term used to account for the different types of evidence or data sources (e.g., quantitative and/or qualitative research, expert opinion, and policy documents) that may be eligible in a scoping review as opposed to only studies. This is not to be confused with *information sources* (see first footnote).

‡ The frameworks by Arksey and O'Malley (6) and Levac and colleagues (7) and the JBI guidance (4, 5) refer to the process of data extraction in a scoping review as data charting.

§ The process of systematically examining research evidence to assess its validity, results, and relevance before using it to inform a decision. This term is used for items 12 and 19 instead of "risk of bias" (which is more applicable to systematic reviews of interventions) to include and acknowledge the various sources of evidence that may be used in a scoping review (e.g., quantitative and/or qualitative research, expert opinion, and policy document).

From: Tricco AC, Lillie E, Zarin W, O'Brien KK, Colquhoun H, Levac D, et al. PRISMA Extension for Scoping Reviews (PRISMAScR): Checklist and Explanation. Ann Intern Med. 2018;169:467–473. doi: 10.7326/M18-0850.

For peer review only - http://bmjopen.bmj.com/site/about/guidelines.xhtml
